# Cardiovascular-kidney-metabolic syndrome: candidate subtypes and genetic risk factors

**DOI:** 10.1186/s12920-026-02315-8

**Published:** 2026-01-31

**Authors:** Hylke C. Donker, Vartika Bisht, Om Prakash Dwivedi, Stefanie Mueller, Dorien Neijzen, Zhihao Ding, Gerton Lunter

**Affiliations:** 1 Department of Epidemiology, University Medical Centre Groningen, University of Groningen, Hanzeplein 1, Groningen, 9713GZ Groningen The Netherlands; 2 Department of Pediatrics, University Medical Centre Groningen, University of Groningen, Hanzeplein 1, Groningen, 9713GZ Groningen The Netherlands; 3 Computational Innovation, Boehringer Ingelheim Pharma GmbH & Co. KG, Birkendorfer Str. 65, Biberach an der Riß, 88400 Baden-Württemberg Germany

**Keywords:** Cardiovascular-kidney-metabolic syndrome, Genome-wide association study, Topic modelling

## Abstract

**Background:**

Cardiovascular-kidney-metabolic (CKM) syndrome is increasingly recognized as a distinct disorder with important implications for health outcomes, but its heterogeneity of presentation and genetic underpinning remains poorly understood. We aimed to identify potential CKM subtypes and their genetic basis by analyzing biomarkers and health outcomes in a large biobank.

**Methods:**

Blood and urine biomarkers from 121,918 participants in the Lifelines cohort were analyzed using topic modelling. Candidate CKM subtypes were operationally defined as blood-urine topics that were simultaneously and positively associated with self-reported kidney disease, type 2 diabetes, and cardiovascular disease. Genome-wide association studies were performed on 52,727 genotyped participants to identify common genetic variants linked to these candidate subtypes.

**Results:**

Five candidate CKM subtypes were identified, each characterized by high levels of blood glucose, uric acid, urea and inflammation biomarkers, but differing in liver enzyme, cholesterol, and glycaemic profiles. Genetic analyses revealed 57 genome-wide significant variants, with the majority (35) not detected in single-biomarker analyses. Most variants were subtype-specific, suggesting that distinct biological pathways contribute to these candidate CKM subtypes.

**Conclusions:**

Our analysis suggests distinct genetic architectures underlying different CKM manifestations and demonstrates that combining biomarkers in disease-relevant constellations improves detection of genetic variants.

**Supplementary Information:**

The online version contains supplementary material available at 10.1186/s12920-026-02315-8.

## Background

In their presidential advisory, the American Heart Association recently defined the cardiovascular-kidney-metabolic (CKM) syndrome as “a health disorder due to connections among heart disease, kidney disease, diabetes and obesity leading to poor health outcomes” [1]. This syndrome is characterized by a combination of haemodynamic impairment, dysfunctional fat reservoirs, inflammation, oxidative stress, insulin resistance, and activation of the renin-angiotensin-aldosterone and sympathetic nervous system [2–6], and is increasingly recognized as a very widespread condition in the US population and elsewhere [7, 8].

While a large number of clinical risk factors for CKM are known [9], the genetic underpinning of CKM syndrome remains unclear [10]. Understanding genetic risk factors could shed light on the syndrome’s heterogeneity and underlying biological pathways, which may help identify therapeutic targets [5, 10]. However, relating CKM syndrome to genetic variation is challenging due to the spectrum of severity and heterogeneous presentation of CKM. This heterogeneity itself is incompletely understood. Although a practical staging model exists that ranks progression across four stages [1], it remains largely unknown whether distinct CKM subtypes exist [10]. A recent work studying CKM in a Chinese cohort of 7,195 participants presented evidence for the existence of at least two subtypes [11]. Here we extend this approach using computational methods to analyze blood and urine biomarkers from a large biobank. Using topic modelling, we identify distinct patterns of biomarkers that may represent CKM subtypes, and relate these to genetic markers. This approach offers three strengths. First, it is objective and data-driven, and allows quantitative assessment of subtype presence on a numerical 0–100% scale, which can be used as a quantitative trait in genetic analyses. Second, by combining multiple biomarkers a more comprehensive measure of disease progression is obtained compared to individual biomarkers, increasing statistical power in subsequent association studies [12, 13]. Third, a technical advantage of the method is that it intrinsically deals with missing data without requiring imputation, increasing the number of samples as well as biomarkers available for analysis, further increasing statistical power.

Here we use this approach on a set of biomarkers measured in blood and urine in the Lifelines cohort to identify 29 biomarker profiles, five of which represent potential CKM subtypes, and we characterize the shared and unique genetic variants that show a signal of association with each of these subtypes.

## Methods

Access to research data of the Lifelines biobank were licensed from Lifelines (project ID ov21_0208) [14]. Lifelines is a multi-disciplinary prospective population-based cohort study examining in a unique three-generation design the health and health-related behaviours of 167,729 persons living in the North of the Netherlands. It employs a broad range of investigative procedures in assessing the biomedical, socio-demographic, behavioural, physical and psychological factors which contribute to the health and disease of the general population, with a special focus on multi-morbidity and complex genetics. Participants who filled out the questionnaire and for whom blood and urine were measured at the baseline assessment were included ($n=121,918$). In addition to baseline information, we also considered questionnaire outcomes from the second assessment (2a, approximately five years after baseline), the first questionnaire from the third assessment (3a, about ten years after baseline) and the second questionnaire from the third assessment (3b, approximately 12 years after baseline). Variables known to be associated with CKM and measured at baseline were included in the analysis. These comprised demographic characteristics, anthropometric data, blood and urine biomarkers, and self-reported disease status (Table S1) [1, 2, 4, 15–18]. Blood measurements included liver enzymes, lipids, glycaemic traits, and inflammatory and fibrotic markers, while urine measurements comprised albumin and creatinine. Follow-up questionnaires assessing self-reported kidney disease, cardiovascular, or type 2 diabetes mellitus (T2DM) incidence from the second and third assessment were also used. Continuous measurements were discretized into five percentile bins (bin thresholds 10%, 30%, 70% and 90% of the empirical distributions stratified by sex), balancing resolution at the tails of the distribution and number of features, except for blood pressure which was categorized as normal, elevated, or hypertensive following clinical guidelines (Supplementary Material S1.1).

To identify blood-urine subtypes, we employed a Bayesian machine learning approach called topic modelling that can handle missing data without imputation (by conditioning on observed data during posterior inference). This method discovers recurring patterns (topics or “profiles”) in the data and scores each participant between 0–100% for each profile, quantifying the contribution of that profile to their combined measurements [19–21]. The optimal number of profiles ($K=32$) was determined by making a 1:2 ratio training-validation split, estimating the generalization performance for different *K*, and finally choosing *K* that gave the best generalization performance (Supplementary Material, section S1.2.1, Fig. S1). After determining the optimal number of components, eight separate Markov chains were run on the full dataset. Model convergence and mixing was confirmed by monitoring the log-likelihood and by comparing within– and between–chain variance of hidden unit entropy, indicating excellent convergence between chains (Supplementary Material, section S1.3, Fig. S2). The profiles from the eight independent Markov chain simulations were matched using the optimal transport method (Supplementary Material, section S1.4). Silhouette scoring and *t*-SNE visualization showed that three out of the 32 topics exhibited poor identifiability and were excluded from further analysis, leaving 29 robust profiles (Fig. S3, Supplementary Material). Samples were then pooled to estimate credible intervals.

Associations between the posterior average of the profiles and demographics and anthropometrics as input (excluding cases with incomplete input), and (self-reported) disease outcomes (per questionnaire, excluded participants with incomplete outcomes) were tested using multivariate linear regression on rank-transformed pattern scores, with Bonferroni correction for multiple testing. Full details are provided in the Supplementary Material (sections S1.2–S1.4).

Candidate CKM subtypes were operationally defined as topics that were simultaneously associated (after multiple testing correction) to self-reported kidney disease, T2DM, and cardiovascular disease (CVD) at the baseline questionnaire.

Genome-wide association studies of common variants located on autosomal regions were carried on on the sub-cohort of self-reported whites (97%) using two models: one adjusted for demographics (age, sex, and smoking) and another additionally adjusted for anthropometrics (body mass index [BMI], waist-hip ratio, weight). These analyses were performed separately for the sub-cohorts genotyped on three different genotyping platforms, and subsequently meta-analyzed to account for batch effects (Supplementary Material S1.5). Given five separate analyses, genome-wide significance was set at $p < 1 \cdot 10^{-8}$. Throughout this work, we report results based on lead variants only (assessed using FUMA, see Supplementary Material S1.5).

## Results and Discussion

The cohort comprised predominantly whites ($>96\%$) with slightly more women than men (72k versus 50k) and $47 \pm 13$ years of age (Table 1). With an average waist-to-hip ratio of $0.9 \pm 0.1$ and $1.0 \pm 0.1$ for women and men, respectively, this is a centrally obese population [6]. As measured by BMI, the majority is either overweight ($39\%$) or obese ($15\%$).

**Table 1:** Participants characteristics of the LifeLines biobank used to construct blood and urine profiles. Mean:$\mu$; standard deviation:$\sigma$

		Missing	Overall	Female	Male
*N*			121,918	72,222	49,696
Age (years),$\mu \, (\sigma )$		0	46.6 (12.8)	46.0 (12.7)	47.4 (12.8)
Ethnicity, *N* (%)	missing		1,437 (1.2)	1,059 (1.5)	378 (0.8)
	Asian		598 (0.5)	376 (0.5)	222 (0.4)
	Black		197 (0.2)	110 (0.2)	87 (0.2)
	other		1,617 (1.3)	1,076 (1.5)	541 (1.1)
	White		118,069 (96.8)	69,601 (96.4)	48,468 (97.5)
Smoker, *N* (%)	missing		125 (0.1)	80 (0.1)	45 (0.1)
	current/recent		23,792 (19.5)	13,248 (18.3)	10,544 (21.2)
	ex		42,066 (34.5)	24,338 (33.7)	17,728 (35.7)
	never		55,935 (45.9)	34,556 (47.8)	21,379 (43.0)
Body mass index (kg$\cdot$m${}^{-2}$), *N* (%)	missing		40 (0.0)	23 (0.0)	17 (0.0)
	normal		54,913 (45.0)	36,216 (50.1)	18,697 (37.6)
	obesity-CI		13,895 (11.4)	8,153 (11.3)	5,742 (11.6)
	obesity-CII		3,305 (2.7)	2,426 (3.4)	879 (1.8)
	obesity-CIII		1,074 (0.9)	869 (1.2)	205 (0.4)
	overweight		47,771 (39.2)	23,798 (33.0)	23,973 (48.2)
	underweight		920 (0.8)	737 (1.0)	183 (0.4)
Waist-hip-ratio (-),$\mu \, (\sigma )$		42	0.9 (0.1)	0.9 (0.1)	1.0 (0.1)
Mass (kg),$\mu \, (\sigma )$		40	79.6 (15.1)	73.9 (13.7)	87.9 (13.2)

### Blood-urine subtypes

**Fig. 1:**
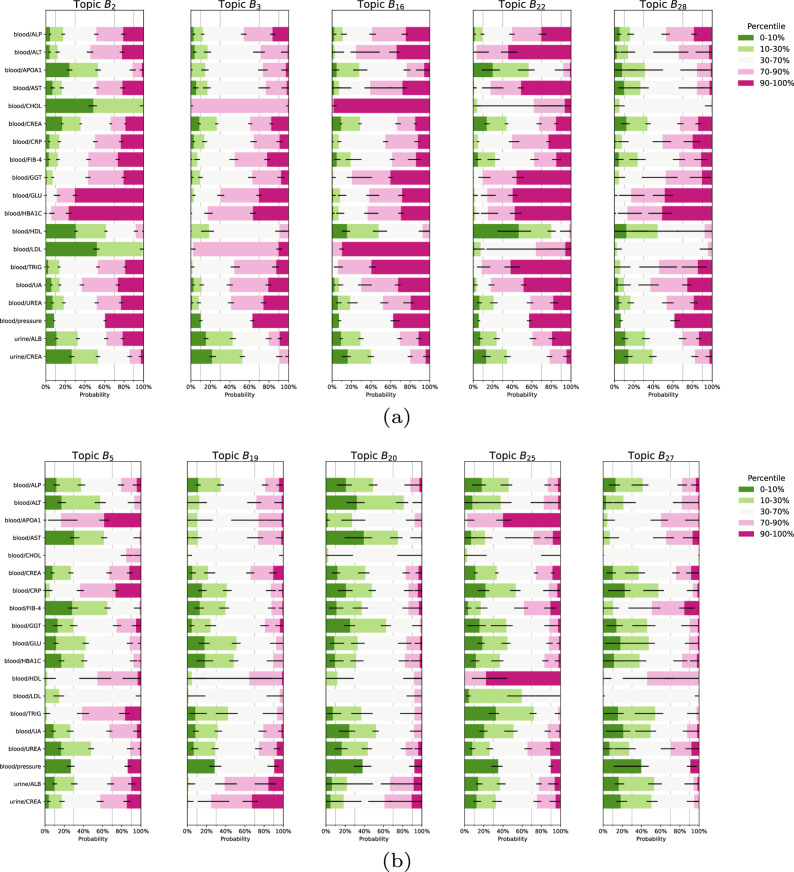
Blood and urine analysis identifies candidate cardiovascular-kidney-metabolic (CKM) subtypes and protective profiles in a population-based cohort. **a** Five blood-urine profiles identified as candidate CKM syndrome subtypes, characterized by positive association with self-reported CKM-related diseases. **b** Five blood-urine profiles identified as CKM-protective, characterized by negative association with self-reported CKM-related diseases. Blood and urine measurements were discretized into five percentile bins of [0–10%, 10–30%,30–70%, 70–90%,90–100%] except for blood pressure (which was discretized as normal, elevated, and hypertension). Error bars indicate 95% credible intervals. Abbreviations: ALB, albumin; ALP, alkaline phosphatase; ALT, alanine aminotransferase; APOA1, apolipoprotein a1; AST, aspartate aminotransferase; CHOL, total cholesterol; CREA, creatinine; CRP, C-reactive protein; FIB-4, fibrosis-4 index; GGT, $\gamma$-glutamyltransferase; GLU, glucose; HBA1C, glycated haemoglobin; HDL, high-density lipoprotein cholesterol; LDL, low-density lipoprotein cholesterol; TRIG, triglyceride; UA, uric acid

**Fig. 2:**
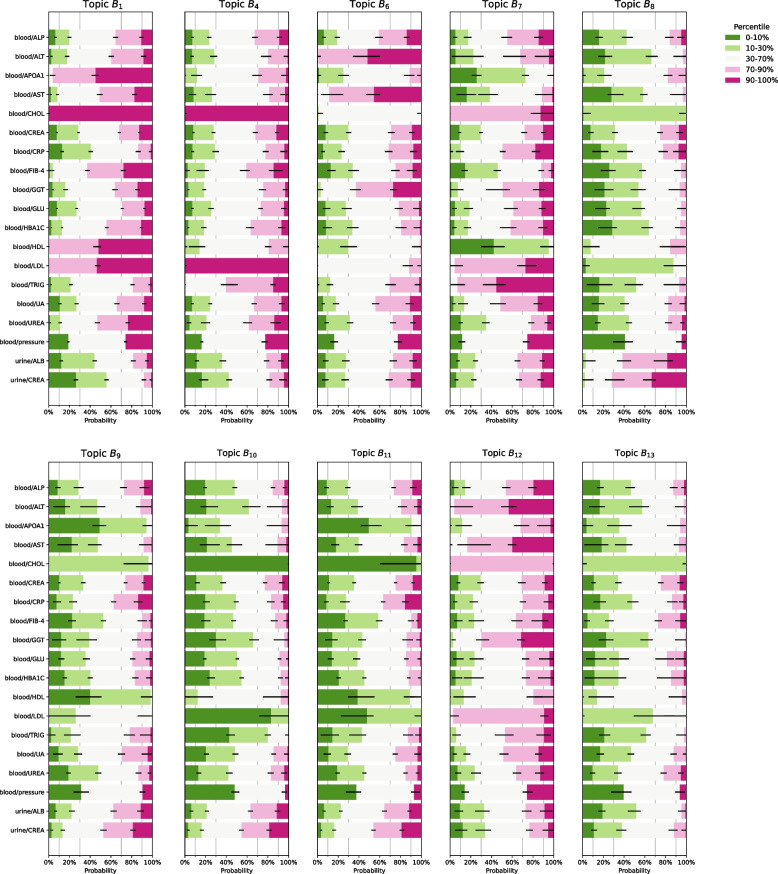
Blood and urine profiles not directly involved in cardiovascular-kidney-metabolic syndrome: B${}_{1}$, B${}_{4}$, B${}_{6}$–B${}_{13}$. Profiles B${}_{2}$, B${}_{3}$ and B${}_{5}$ are listed Fig. 1. Blood and urine measurements were discretized into five percentile bins of [0–10%, 10–30%,30–70%, 70–90%,90–100%] except for blood pressure (which was discretized as normal, elevated, and hypertension). Error bars indicate 95% credible intervals. Abbreviations: ALB, albumin; ALP, alkaline phosphatase; ALT, alanine aminotransferase; APOA1, apolipoprotein a1; AST, aspartate aminotransferase; CHOL, total cholesterol; CREA, creatinine; CRP, C-reactive protein; FIB-4, fibrosis-4 index; GGT, $\gamma$-glutamyltransferase; GLU, glucose; HBA1C, glycated haemoglobin; HDL, high density lipoprotein cholesterol; LDL, low density lipoprotein cholesterol; TRIG, triglyceride; UA, uric acid

**Fig. 3:**
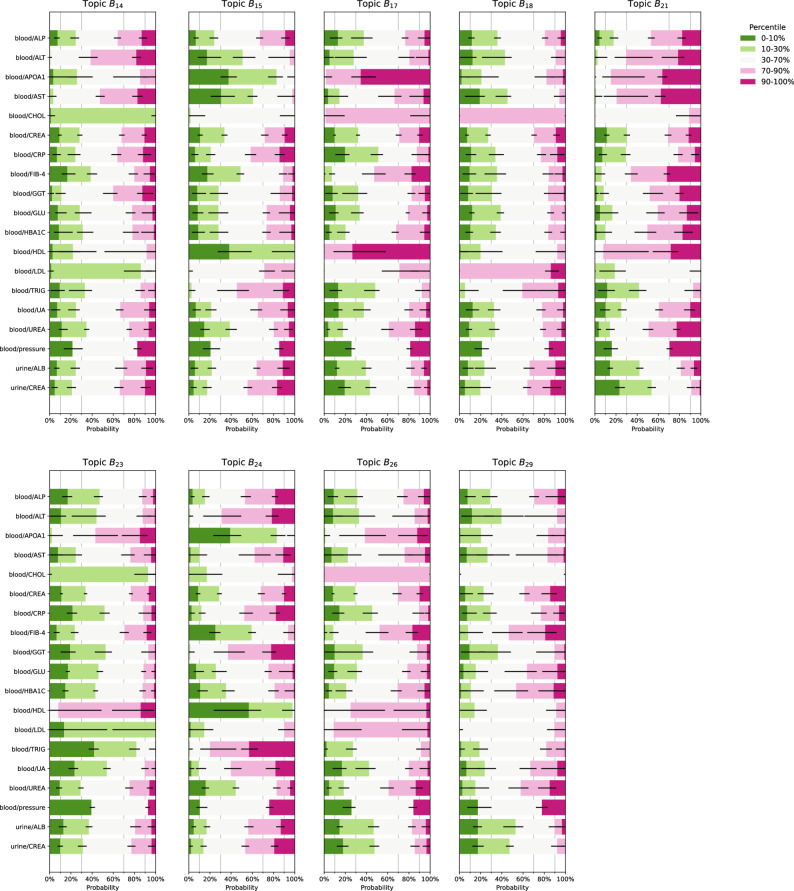
Continuation of Fig. 2: blood and urine profiles not directly involved in cardiovascular-kidney-metabolic syndrome: B${}_{14}$, B${}_{15}$, B${}_{17}$,B${}_{18}$, B${}_{21}$, B${}_{23}$, B${}_{24}$, B${}_{26}$ and B${}_{29}$. Profiles B${}_{16}$, B${}_{19}$, B${}_{20}$, B${}_{22}$, B${}_{25}$, B${}_{27}$, and B${}_{28}$ are listed in Fig. 1. Blood and urine measurements were discretized into five percentile bins of [0–10%, 10–30%,30–70%, 70–90%,90–100%] except for blood pressure (which was discretized as normal, elevated, and hypertension). Error bars indicate 95% credible intervals. Abbreviations: ALB, albumin; ALP, alkaline phosphatase; ALT, alanine aminotransferase; APOA1, apolipoprotein a1; AST, aspartate aminotransferase; CHOL, total cholesterol; CREA, creatinine; CRP, C-reactive protein; FIB-4, fibrosis-4 index; GGT, $\gamma$-glutamyltransferase; GLU, glucose; HBA1C, glycated haemoglobin; HDL, high density lipoprotein cholesterol; LDL, low density lipoprotein cholesterol; TRIG, triglyceride; UA, uric acid

After determining the optimal number of components ($K=32$, Fig. S1, Supplementary Material), analysis of blood and urine biomarkers pertaining to CKM syndrome revealed 29 robust profiles (Figs. 1, 2, 3 and S3, Supplementary Material).

**Fig. 4:**
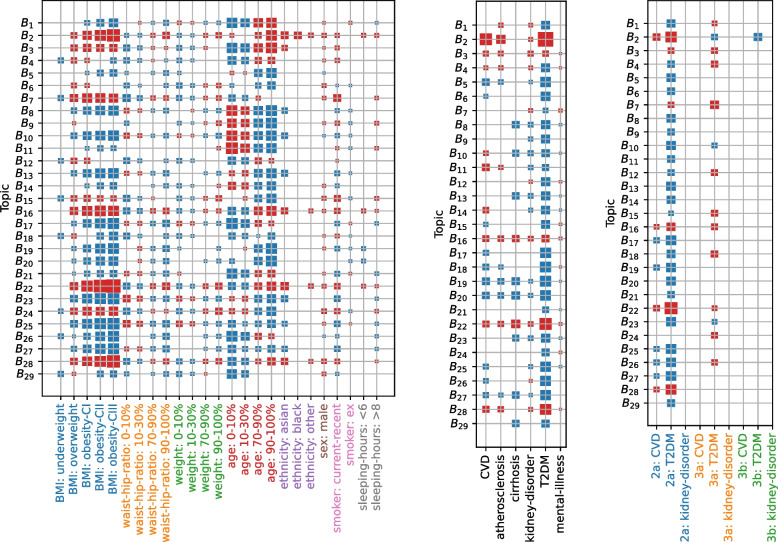
Profiles B${}_2$, B${}_3$, B${}_{16}$, B${}_{22}$, and B${}_{28}$ are associated to pre-existing CKM and incidence of T2DM and CVD. **a** Association between demographics and anthropometrics with topic loading ($N=108,696$ cases). **b** Association between self-reported disease presence during the first Lifelines baseline assessment and topic loading ($N=121,764$ cases). **c** Associations between self-reported incidence of disease based on questionnaires from the lifelines 2nd assessment (2a, $N=7,050$ cases), third assessment (3a, $N=1,200$ cases), and a follow-up questionnaire of the third assessment (3b, $N=459$ cases) and topic loading. The size of the red (blue) square scales $\propto \sqrt{|\beta |}$ with multivariate linear regression coefficient $\beta$ and marks a positive (negative) statistical association (*t*-test with Bonferroni multiple testing correction). Abbreviations: BMI, body mass index; CVD, cardiovascular disease; T2DM: type 2 diabetes mellitis

Of these, five profiles (B${}_2$, B${}_3$, B${}_{16}$, B${}_{22}$, and B${}_{28}$) emerged as distinct CKM syndrome subtypes (Fig. S3), based on their simultaneous significant and positive associations with pre-existing CVD, kidney disease, and T2DM (Bonferroni corrected *t*-test, Fig. 4). Common themes among these subtypes were high levels of glucose and glycated haemoglobin (HBA1C), often in the highest decile corresponding to pre-diabetes^1^ or hyperglycaemia,^2^ combined with high levels of uric acid and urea in blood as well frequent occurrence of hypertension and infrequently observed normal blood pressure (Fig. 1a). CKM-associated profiles also showed higher concentrations of C-reactive protein (CRP) indicative of inflammation.

The CKM-associated profiles differed markedly across the glycaemic, lipid, and liver enzyme axes. For instance, B${}_2$ describes a glycaemic high, cholesterol low subtype, with cholesterol (CHOL) and low-density lipoprotein (LDL) concentrations exclusively in the bottom 30% corresponding to desirable total cholesterol^3^ and optimal or near optimal levels of LDL.^4^ Concurrently, the profile is enriched for low levels of high-density lipoprotein (HDL) and apolipoprotein a1 (APOA1) concentrations at the bottom 30% of the population. B${}_2$ showed consistently higher liver enzyme levels [i.e., alanine aminotransferase (ALT), aspartate aminotransferase (AST), alkaline phosphatase (ALP), and $\gamma$-glutamyltransferase (GGT)]. In addition, B${}_2$ over-represents high levels of fibrosis-4 index (FIB-4) together with low levels creatinine in urine (CREA, which estimates kidney glomerular filtration rate [24]).

By contrast, B${}_3$ is primarily driven by CHOL and LDL levels in the 70–90% quantile range, corresponding to borderline high LDL^5^ and total cholesterol^6^ to high LDL^7^ and high total cholesterol.^8^ HDL and APOA1 levels, and to a lesser extent the liver enzymes, are around the population median (the 30–70% percentile). Similar to B${}_2$, B${}_3$ over-represents low levels of CREA.

Topic $B_{16}$ describes a dyslipidemia^9^ and elevated liver enzyme blood phenotype. Total and LDL cholesterol levels rank exclusively at the top decile, and triglycerides above the 70% percentile of the cohort, and an enrichment for low HDL cholesterol levels. Concurrently, liver enzymes are consistently at the far end of the spectrum.

Profile B${}_{22}$ is an exaggeration of B${}_{16}$ without the hypercholesterolemic component. Profile B${}_{22}$ is primarily driven by the liver enzyme and glycaemic axis, with substantial top decile over-representation. Despite uncertainty on the LDL and total cholesterol levels, there was a slight, but consistent, enrichment for cholesterol levels at the bottom 30%. Triglycerides levels come predominantly from the top decile, like B${}_{16}$, and together with lower concentrations of HDL and APOA1.

Finally, B${}_{28}$ is characterized by cholesterol and LDL levels fall with near certainty in the 30–70% percentile of the population [largely overlapping desirable total, and near optimal LDL (2.59−3.34 mM), cholesterol].

Our findings qualitatively match the MetS and CVD profiles reported earlier by Zeng & Xiao. [11] (although differences in input variables and cut-off values prevents us from making a one-to-one correspondence).

We also identified five CKM-protective profiles (B${}_5$, B${}_{19}$, B${}_{20}$, B${}_{25}$, and B${}_{27}$), by simultaneous significant negative associations with pre-existing CVD, kidney disease, and T2DM (Bonferroni corrected *t*-test, Fig. 1b). The disinclination for extreme levels of CHOL and LDL (both the lowest and highest 30%)—which we interpret as homeostasis—appeared a unifying theme of CKM resilience. Besides these common factors, these profiles also showed clear differences. In B${}_5$, the CRP level distribution was a mirror opposite of the FIB-4 score, with increasing probability of getting both higher CRP and lower FIB-4 scores. At the same, there was also a consistent shift towards higher levels of TRIG and APOA1. Profile B${}_{20}$ is a liver low, lipid central, profile. Extreme levels (top or bottom 30%) were attenuated across the entire lipid compartment, as well as for APOA1. Further, this profile was marked by a shift towards lower (bottom 30% of the population) liver enzyme levels. In contrast, B${}_{25}$ describes a “good cholesterol”-dominating blood profile. Both APOA1 and HDL levels were over representatively high (mostly in the top decile of the cohort). This was in combination with reduced levels of TRIG. Finally, B${}_{19}$ and B${}_{27}$ are urine high and low profiles, respectively. Compared to the entire population, both ALB and CREA urine levels were higher for B${}_{19}$, while the opposite was seen for B${}_{27}$, in line with urine albumin-to-creatinine ratio as an important indicator of kidney function.

### Epidemiology of subtypes

Most participants showed minimal loading on the five candidate subtypes, with median proportions (of the posterior average) ranging from 0.25 to 0.40% (Table S6). While participants typically showed loading on several subtypes (Fig. S6, Supplementary Material), we defined subtype-positive cases using a topic loading threshold of $50\%$. Using this definition, prevalence for the topics ranged from 1.26% (B${}_{28}$, 1,541 cases) to 2.46% (B${}_{16}$, 3,003 cases; [Table S6]). The overall prevalence of CKM syndrome, defined as the sum of topic loadings across the five subtypes meeting the $50\%$ threshold, was 11.5% of the population (13,987 cases). Sensitivity analysis revealed that lowering the threshold from 50% to 40% or 30% increased prevalence from 11.5% to 13.7% and 16.4%, with a similar trend for individual subtypes (Table S7).

All five subtypes were linked to current or recent smokers and the male gender (Fig. 4). While the CKM subtypes were primarily driven by White participants (Table S6), we nevertheless found that Asian participants scored on average higher on all CKM subtypes than White participants, in agreement with previous reports [1]. Other minorities were also associated to CKM profiles (with Blacks associated to B${}_2$, and “other” associated to all but B${}_3$). As expected, topic loading increased with increasing BMI and waist-to-hip ratio (WHR). Older age (70–90% and 90–100% percentile buckets) were associated with higher CKM topic proportions, and vice versa for younger age, with the exception of glycaemic high, low cholesterol, subtype B${}_{2}$, which showed a significant prevalence also in the 10% youngest group ($\le 28$ years old women, and $\le 30$ men).

Sleep duration and sleep disorder are known risk factors for metabolic and CKM syndrome [1, 11, 27, 28]. From the five subtypes, only B${}_3$ did not significantly associate with $>8$ hours of sleep, while B${}_2$, B${}_{16}$, and B${}_{22}$ were linked to both short and long self-reported sleep duration, consistent with a previously reported U-shaped risk profile for sleep duration [28]. All candidate subtypes except B${}_2$ were associated with self-reported pre-existing mental disorder (defined in Sec. S1.1, Supplementary Material), a known risk factor for CKM [1, 29]. Liver cirrhosis at baseline was associated with B${}_{16}$ and B${}_{22}$, in line with a shift towards high liver enzymes in those profiles (Fig. 1). In terms of incidence, all candidate profiles were positively associated with incidence of T2DM, and all but B${}_3$ were associated with incidence of CVD in the second assessment (about five years after the baseline assessment). Surprisingly, none of the topics (out of all 29) were significantly associated with incidence of kidney disorder (at the second or third assessments), which we attributed to the strict multiple testing correction.

Finally, we observed anti-correlation between B${}_2$ and T2DM at the third assessment (during the first questionnaire [3a] about 10 years after baseline, and the follow-up questionnaire [3b] about 12 years after baseline). We speculate that this might be due to B${}_2$’s enrichment for the youngest participants ($\le 30$ years) that remain T2DM-free after a proportion of elderly participants have progressed to T2DM.

Profiles protective of CKM largely showed the same relations but where the direction of effect was reversed (Fig. 4). One notable exceptions is that both B${}_{27}$ and B${}_{29}$ were mostly associated with women between 40–51 years of age and 41–53 years for men, rather than a younger cohort.

### Genome-wide association studies

**Fig. 5:**
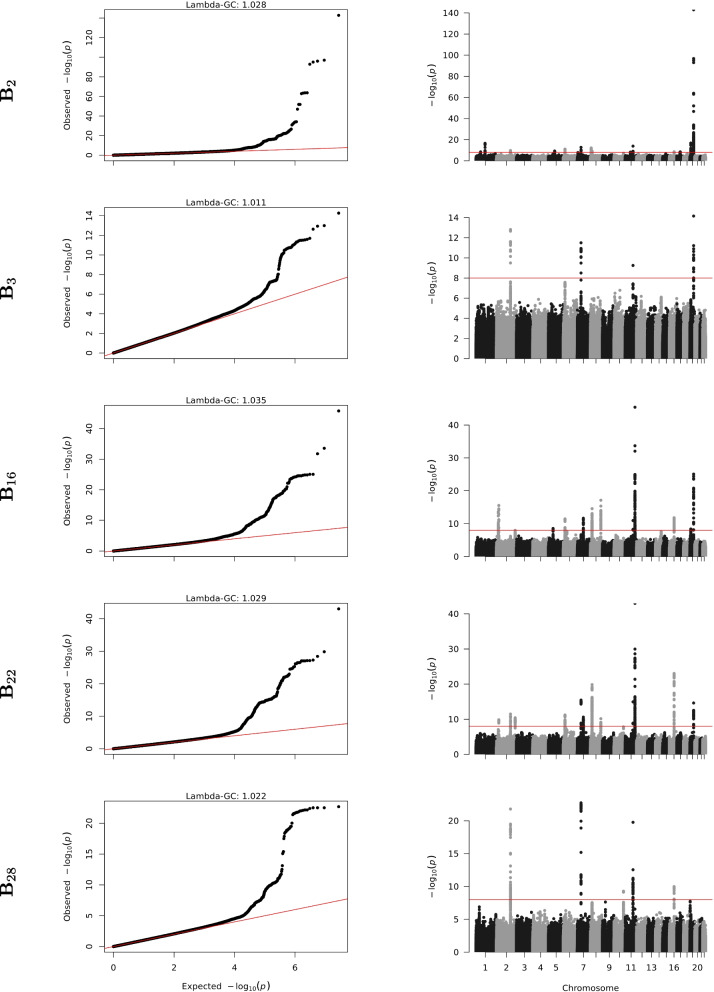
Meta-analysis of unrelated individuals identifies shared and unique genetic variants underpinning five candidate CKM subtypes (B${}_{2}$, B${}_{3}$, B${}_{16}$, B${}_{22}$, and B${}_{28}$). The quantile-quantile plots (and genomic inflation factor $\lambda _{\text {GC}}$) show no clear signs of *p*-value inflation (left) and Manhattan plots show significant hits in strong linkage-disequilibrium (right) indicative of biology, rather than technical artefacts. All analyses were adjusted for age, sex (male versus female), smoking status (current/recent, ex-, and never smokers), body mass index, waist-to-hip ratio, and weight. The red diagonal line in the quantile-quantile plot is the expected distribution under the null hypothesis; the red horizontal line in the Manhattan plot marks the significance threshold

To determine the genetic predisposition for the different subtypes, five separate genome-wide association study (GWAS) meta-analyses were conducted (one per candidate subtype). Out of 52,994 included participants (Table S3, Supplementary Material), genotypes of 52,727 participants passed all quality controls (6,114 samples genotyped on the Illumina CytoSNP chip, 23,956 on the Affymetrix microarray, and 22,657 on the Infinium Global Screening Array) and were meta-analyzed. Across the 5 candidate subtypes, we found 57 genome-wide significant associations (Fig. 5, Tables 2 and 3). Most variants (31 out of 57) were detected for B${}_2$, of which 27 were unique to B${}_2$, and only four variants were found shared with other subtypes. This was followed with B${}_{16}$ with 17 leads (of which nine were private to B${}_{16}$), and B${}_{22}$ with 15 genome-wide significant leads (of which six unique). For B${}_{28}$ and B${}_3$ only four (one private) and six (four private) leads were found, respectively.

**Table 2:** Genome-wide significant single nucleotide variant leads per candidate cardiovascular-kidney-metabolic syndrome subtype (namely, B${}_2$, B${}_3$, B${}_{16}$, B${}_{22}$, and B${}_{28}$) on chromosomes 1, 2, 5–8, 10, and 11 (see Table 3 for the rest). Abbreviations: Chr, chromosome; EA, effect allele; HGVS, Human Genome Variation Society nomenclature; MAF, minor allele frequency; NEA, non-effect allele; Pos: genomic position on the human reference genome GRCh37; rsID, reference single nucleotide variant cluster identifier; SE, standard error

rsID	Subtype	Chr	Pos	EA/NEA	HGVS	MAF	$\beta$(SE)	$\log _{10} p$	Nearest gene
rs12117661^a^	B${}_2$	1	55487346	G/C	NC_000001.10:g.55487346C>G	0.231	0.04 (0.007)	−8.42	BSND
rs583104^a^	B${}_2$	1	109821307	G/T	NC_000001.10:g.109821307G>T	0.223	0.058 (0.007)	−16.56	PSRC1
rs1041968^a^	B${}_{16}$	2	21232804	G/A	NC_000002.11:g.21232804G>A	0.442	−0.042 (0.006)	−13.53	APOB
rs533617^a^	B${}_{16}$	2	21233972	C/T	NC_000002.11:g.21233972T>C	0.049	−0.076 (0.012)	−9.6	APOB
rs1260326	B${}_{16}$	2	27730940	C/T	NC_000002.11:g.27730940T>C	0.41	−0.047 (0.006)	−15.51	GCKR
	B${}_{22}$						−0.036 (0.006)	−9.85	
rs13431652	B${}_2$	2	169753415	C/T	NC_000002.11:g.169753415T>C	0.311	−0.043 (0.007)	−9.75	SPC25
	B${}_{22}$						−0.043 (0.006)	−11.48	
	B${}_{28}$						−0.063 (0.006)	−21.79	
rs853787^a^	B${}_3$	2	169802252	G/T	NC_000002.11:g.169802252G>T	0.353	−0.042 (0.006)	−12.82	ABCB11
rs2943653^a^	B${}_{16}$	2	227047771	C/T	NC_000002.11:g.227047771C>T	0.342	−0.034 (0.006)	−8.01	AC068138.1
rs2943634^a^	B${}_{22}$	2	227068080	C/A	NC_000002.11:g.227068080A>C	0.348	0.037 (0.006)	−10.41	AC068138.1
rs71624138^a^	B${}_{16}$	5	55870395	G/A	NC_000005.9:g.55870395G>A	0.128	−0.056 (0.009)	−8.57	AC022431.2
rs6882842^a^	B${}_2$	5	74651909	G/A	NC_000005.9:g.74651909G>A	0.408	0.036 (0.006)	−9.25	HMGCR
rs35261542	B${}_{16}$	6	20675792	C/A	NC_000006.11:g.20675792C>A	0.279	−0.043 (0.006)	−11.44	CDKAL1
	B${}_{22}$						−0.041 (0.006)	−11.22	
rs6931514^a^	B${}_2$	6	20703952	G/A	NC_000006.11:g.20703952A>G	0.279	0.045 (0.007)	−11.07	CDKAL1
rs2908290^a^	B${}_{28}$	7	44216137	G/A	NC_000007.13:g.44216137G>A	0.408	−0.036 (0.006)	−8.96	GCK
rs917793^a^	B${}_2$	7	44245853	T/A	NC_000007.13:g.44245853A>T	0.178	0.06 (0.008)	−12.75	YKT6
	B${}_3$						0.053 (0.008)	−11.5	
rs2908282	B${}_{28}$	7	44248828	G/A	NC_000007.13:g.44248828G>A	0.178	−0.074 (0.007)	−22.73	YKT6
rs878521^a^	B${}_{22}$	7	44255643	G/A	NC_000007.13:g.44255643G>A	0.254	−0.05 (0.006)	−15.44	CAMK2B
rs62465144^a^	B${}_{22}$	7	72883106	C/T	NC_000007.13:g.72883106T>C	0.189	−0.046 (0.007)	−10.13	BAZ1B
rs11974409^a^	B${}_{16}$	7	72989390	G/A	NC_000007.13:g.72989390A>G	0.186	−0.048 (0.007)	−11.62	TBL2
rs11786900^a^	B${}_2$	8	9239958	G/C	NC_000008.10:g.9239958G>C	0.076	−0.072 (0.01)	−12.16	RP11-115J16.1, RP11-115J16.2
rs268	B${}_2$	8	19813529	G/A	NC_000008.10:g.19813529A>G	0.014	0.107 (0.017)	−9.21	LPL
	B${}_{16}$						0.096 (0.016)	−8.47	
	B${}_{22}$						0.132 (0.016)	−16.21	
rs286^a^	B${}_2$	8	19815256	T/A	NC_000008.10:g.19815256A>T	0.111	−0.066 (0.011)	−8.66	LPL
rs9644636^a^	B${}_{22}$	8	19824896	G/T	NC_000008.10:g.19824896T>G	0.272	0.036 (0.006)	−8.01	LPL
rs115849089^a^	B${}_{16}$	8	19912370	G/A	NC_000008.10:g.19912370G>A	0.145	0.065 (0.008)	−14.54	AC100802.3
	B${}_{22}$						0.075 (0.008)	−19.84	
rs28601761	B${}_{16}$	8	126500031	G/C	NC_000008.10:g.126500031C>G	0.408	−0.048 (0.006)	−17.11	RP11-136O12.2
	B${}_{22}$						−0.035 (0.005)	−10.16	
rs34872471^a^	B${}_{28}$	10	114754071	C/T	NC_000010.10:g.114754071T>C	0.315	0.039 (0.006)	−9.28	TCF7L2
rs174564	B${}_2$	11	61588305	G/A	NC_000011.9:g.61588305A>G	0.353	0.036 (0.006)	−8.25	FADS2, FADS1
rs10830963	B${}_2$	11	92708710	G/C	NC_000011.9:g.92708710C>G	0.288	0.052 (0.007)	−13.89	MTNR1B
	B${}_3$						0.039 (0.006)	−9.25	
	B${}_{16}$						0.043 (0.006)	−10.92	
	B${}_{22}$						0.049 (0.006)	−14.94	
	B${}_{28}$						0.06 (0.006)	−19.77	
rs141414463	B${}_{16}$	11	116598315	C/T	NC_000011.9:g.116598315C>T	0.012	−0.102 (0.017)	−8.83	BUD13
rs964184	B${}_{16}$	11	116648917	G/C	NC_000011.9:g.116648917G>C	0.162	0.116 (0.008)	−45.43	ZNF259
	B${}_{22}$						0.11 (0.008)	−42.98	

^a^Variants that did not reach significance (in this cohort) in any of the blood molecules, urine molecules, and FIB-4 index when analyzed individually

**Table 3:** Continuation of Table 2. Abbreviations: Chr, chromosome; EA, effect allele; HGVS, Human Genome Variation Society nomenclature; MAF, minor allele frequency; NEA, non-effect allele; Pos: genomic position on the human reference genome GRCh37; rsID, Reference single nucleotide variant cluster identifier; SE, standard error.

rsID	Subtype	Chr	Pos	EA/NEA	HGVS	MAF	$\beta$(SE)	$\log _{10} p$	Nearest gene
rs56156922^a^	B${}_{28}$	16	56987369	C/T	NC_000016.9:g.56987369T>C	0.293	−0.039 (0.006)	−9.98	AC012181.1
rs1800775	B${}_{16}$	16	56995236	C/A	NC_000016.9:g.56995236C>A	0.488	0.039 (0.006)	−11.78	CETP
rs118146573^a^	B${}_{22}$	16	57000938	G/A	NC_000016.9:g.57000938G>A	0.122	−0.068 (0.008)	−15.55	CETP
rs7205804^a^	B${}_{22}$	16	57004889	G/A	NC_000016.9:g.57004889G>A	0.427	0.054 (0.005)	−23.03	CETP
rs11076175	B${}_2$	16	57006378	G/A	NC_000016.9:g.57006378A>G	0.206	0.047 (0.008)	−8.56	CETP
rs14050^a^	B${}_2$	17	37828072	C/T	NC_000017.10:g.37828072C>T	0.314	0.037 (0.006)	−8.11	PGAP3
rs112259268^a^	B${}_2$	17	41874745	C/A	NC_000017.10:g.41874745C>A	0.035	−0.095 (0.016)	−8.44	MPP3
rs112107114^a^	B${}_{16}$	19	11190074	G/A	NC_000019.9:g.11190074G>A	0.111	0.05 (0.008)	−8.38	LDLR
rs17248720^a^	B${}_2$	19	11198187	C/T	NC_000019.9:g.11198187C>T	0.108	−0.077 (0.009)	−16.77	LDLR
rs188099946	B${}_2$	19	45189605	C/T	NC_000019.9:g.45189605C>T	0.015	−0.138 (0.024)	−8.12	CTB-171A8.1
rs151330717	B${}_2$	19	45196964	G/A	NC_000019.9:g.45196964G>A	0.012	−0.192 (0.025)	−14.05	CTB-171A8.1
rs11881756	B${}_2$	19	45220896	C/T	NC_000019.9:g.45220896T>C	0.102	0.072 (0.011)	−10.02	CTB-171A8.1
rs2965169^a^	B${}_2$	19	45251156	C/A	NC_000019.9:g.45251156A>C	0.437	0.055 (0.007)	−15.83	BCL3
rs2967668	B${}_2$	19	45302951	G/A	NC_000019.9:g.45302951A>G	0.126	0.056 (0.01)	−8.08	CBLC
rs10402271^a^	B${}_2$	19	45329214	G/T	NC_000019.9:g.45329214T>G	0.345	−0.05 (0.006)	−15.3	BCAM
rs111371860	B${}_2$	19	45345787	T/A	NC_000019.9:g.45345787A>T	0.076	0.132 (0.013)	−23.43	PVRL2
rs2927472^a^	B${}_2$	19	45349369	C/T	NC_000019.9:g.45349369T>C	0.135	−0.083 (0.008)	−22.39	PVRL2
rs6857^a^	B${}_3$	19	45392254	C/T	NC_000019.9:g.45392254C>T	0.165	−0.056 (0.007)	−14.16	PVRL2, CTB-129P6.4
	B${}_{16}$						−0.075 (0.007)	−25.06	
	B${}_{22}$						−0.055 (0.007)	−14.62	
rs11668327	B${}_2$	19	45398633	G/C	NC_000019.9:g.45398633G>C	0.156	−0.078 (0.008)	−20.2	TOMM40
rs7412	B${}_2$	19	45412079	C/T	NC_000019.9:g.45412079C>T	0.063	−0.278 (0.011)	−142.97	APOE
rs12721051	B${}_2$	19	45422160	G/C	NC_000019.9:g.45422160C>G	0.198	−0.059 (0.008)	−14.45	APOC1
rs157595^a^	B${}_{16}$	19	45425460	G/A	NC_000019.9:g.45425460A>G	0.397	0.038 (0.006)	−10.16	APOC1
rs71352239^a^	B${}_2$	19	45429543	C/T	NC_000019.9:g.45429543C>T	0.301	0.05 (0.007)	−13.81	APOC1P1
rs4263041	B${}_2$	19	45438643	G/A	NC_000019.9:g.45438643A>G	0.288	0.044 (0.008)	−8.19	APOC1P1
rs117261169	B${}_2$	19	45491032	C/T	NC_000019.9:g.45491032C>T	0.011	−0.214 (0.027)	−14.93	CLPTM1
rs140912273^a^	B${}_2$	19	45507696	G/A	NC_000019.9:g.45507696G>A	0.014	−0.157 (0.025)	−9.39	RELB
rs3178166^a^	B${}_2$	19	45594170	G/A	NC_000019.9:g.45594170A>G	0.463	0.036 (0.006)	−8.58	GEMIN7, MARK4

^a^Variants that did not reach significance (in this cohort) in any of the blood molecules, urine molecules, and FIB-4 index when analyzed individually

Interestingly, although GWAS on the individual blood and urine biomarkers yielded 742 lead SNVs in total, the majority of the lead SNVs resulting from the GWAS on CKM topics (35 out of 57 variants) were not detected in any of these individual biomarker GWAS. These findings are consistent with a previous study focusing on topics derived from ICD10 codes [12, 13], and indicate that topic modelling can be a useful complementary analysis to detect additional genetic risk factors for a set of related traits.

All of the 57 significant associations were previously reported in the all-phenotype GWAS catalogue [30] or scientific literature. Except for two associations, all have previously been previously reported in the GWAS catalogue as associations with one of the underlying blood and urine measurements used to create the profiles, confirming that these are true associations. Two associations not previously linked to CKM-related blood and/or urine biomarkers, rs111371860 and rs4263041, are within 93 kb of each other (linkage disequilibrium $R^2=0.0134$) and were both previously linked to Alzheimer disease [31], which is consistent with previous reports of adiposity as a causal factor in cognitive decline [32–34]. Since predisposition to obesity is strongly genetically determined [35], we re-analyzed the data without adjusting for BMI, WHR, and weight and found largely the same results, with a total of 52 lead single nucleotide variants (SNVs) reaching genome-wide significance (Tables S8 and S9, Supplementary Material) in strong linkage with lead SNVs identified in the GWAS with adjustment for BMI, WHR, and weight (Fig. S7, Supplementary Material).

**Fig. 6:**
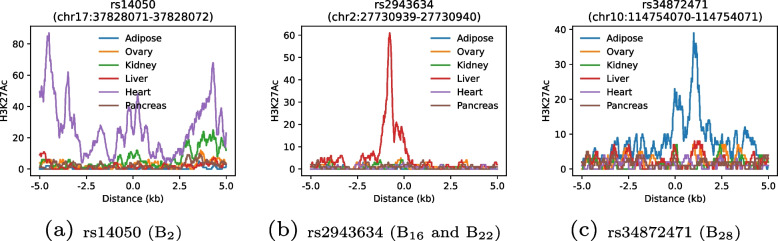
Tissue specificity of selected genetic variants underlying candidate CKM subtypes. Each panel shows H3K27Ac histone modification (indicative of gene activity) $\pm 5$ kb around the variant’s position for different tissue types. Panels display the genomic regions surrounding: **a** rs14050 (associated with B_2_); **b** rs2943634 (associated with subtypes B_16_ and B_22_) **c**. rs34872471 (associated with subtype B_28_)

Further confirmation of the biological plausibility of the identified hits and candidate subtypes was provided by overrepresentation analysis of the nearest genes. All candidate subtypes except for B${}_{28}$ were enriched for cholesterol metabolism, while variants underlying B${}_{28}$ were involved in biological processes such as carbohydrate and glucose metabolism (Fig. S10, Supplementary Material).

To probe tissue specificity, we estimated the amount of H3K27Ac histone modification $\pm 5$ kb around the lead SNVs (indicating gene activity) in adipose, kidney, liver, heart, and pancreas tissue with ovary tissue as a control. While not all SNVs had distinguishing H3K27Ac histone modification patterns (Figs. S8 & S9, Supplementary Material), there were several variants that showed clear tissue specificity (Fig. 6). For example, histones near rs14050 (linked to B${}_2$) were primarily modified in heart cells, while rs2943634 (contributing to both B${}_{16}$ and B${}_{22}$) was strongly peaked in liver cells, whereas histone modification near B${}_{28}$ hit rs34872471 was unique to adipose tissue. All of these three SNVs would’ve been missed in individual biomarker GWAS.

### External Validation in UK Biobank

Next, we constructed polygenic risk scores (PRS) for each candidate subtype using the identified hits from our internal analysis. We then used these PRS to externally validate the subtypes and their associated genetic risk factors within the UK Biobank using diagnoses based on in-hospital records and causes of death (Sec. S1.6.1, Supplementary Material), rather than self-reported disease presence. The goal of this validation was to determine if our subtype PRS could predict CKM diagnoses in an independent cohort, which would confirm the authenticity of our findings.

After selecting participants from the first or second UK Biobank assessment and excluding those unsuitable for genotype analysis (Sec. S1.6.2, Supplementary Material), our final cohort included 333,938 individuals. Three of the five candidate subtypes (B${}_{16}$, B${}_{22}$, and B${}_{28}$) were found to be positively and simultaneously associated with cardiovascular disease, kidney disease, and type 2 diabetes diagnoses (*t*-test, $N=333,938$; see Fig. S11, Supplementary Material), independently confirming their link to CKM. These associations remained significant regardless of whether the PRS was constructed from GWAS results adjusted for obesity-related factors (Fig. S11, Supplementary Material). Association of B${}_2$ and B${}_3$ with kidney disease did not reach significance. Surprisingly, the direction of association between $B_2$ and cardiovascular disease was reversed than expected; the reason for this latter observation is currently unclear. We speculate that this might be a false positive, rather than a systematic effect related to differences in characteristics (such as age) between the cohorts.

## Strengths and Limitations

A strength of our Bayesian machine learning-based approach is that the profiles are largely objective and data-driven. The Bayesian approach allowed us to account for uncertainty, mitigating many sources of bias that often affect other approaches. A large number of participants could be included because the model could be trained on data with partially missing observations, in contrast to deep learning-based representations [36–39], without resorting to imputation, albeit at the expense of some posterior uncertainty. This allowed us to include liver enzymes which were measured in less than half of the included participants (50,224 in total, or 41.2%), and C-reactive protein which was available for one in every three participants (42,056 cases, or 34.5%). Finally, we demonstrated that this approach improves power to detect variants that related to constellations of traits, in line with earlier findings [12, 13].

A limitation of this work is that rather than on doctor’s diagnoses, the link to CKM is based on self-reported disease presence and incidence, which is known to lead to underreporting [40–42]. Nevertheless, we were able to indirectly confirm the involvement of CKM in three of the five subtypes. This was achieved through the association between each subtype’s PRS and CKM disease diagnoses—rather than self-reported data—in an independent cohort (UK Biobank). Further, while conditions like chronic kidney disease and hypertension are often underdiagnosed in clinical settings [43], the candidate CKM subtypes, being derived directly from blood and urine measurements in a population-based cohort, remain objective and are not influenced by such diagnostic biases. Potential biases are thus primarily driven by biobank population composition (overrepresentation of older and female participants compared to the general population [44]).

Second, our subtypes were not externally replicated in other populations.

Third, medication effects on blood-urine measurements are superimposed on to the profiles, since no information was available to discount for medication usage. For example, B${}_2$ is enriched for people under $\le 30$ years of age, and we speculate that B${}_2$ captures both young people with intrinsic low cholesterol as well as an aged population with cholesterol-lowering medication. Fourth, we primarily recapitulate existing knowledge rather than discover the involvement of previously unidentified biomarkers. This was by design: to aid the identification of CKM subtypes, we pre-selected blood and urine biochemicals that were previously known to be involved in CKM [1, 2, 4, 15–18]. Fifth, our GWAS included White participants only to prevent false discoveries that may result from inflated *p*-values due to unaccounted population substructure and related confounding. Ideally, one would also include Asians given predisposition for CKM in the Asian population (although there is some dispute in the literature whether this predisposition is directly related to genetic variants [5] or mediated by differences in body composition profiles for varying BMI [10]).

## Conclusion

Understanding the heterogeneity and genetic underpinning of cardiovascular-kidney-metabolic (CKM) syndrome are two major outstanding problems [10]. Here, we report on five candidate CKM subtypes that were identified by topic modelling of blood and urine biomarker profiles. These profiles share a common constellation of high blood sugars, uric acid, urea and inflammation biomarkers, while they differed along the liver enzyme, cholesterol, and glycaemic compartments. The genetics underlying the CKM candidate subtypes recapitulate known genetic variants previously implicated in CKM-relevant blood and urine biomarkers, some of which were unique to subtypes, and some of which were shared. Our analysis show that topic modelling reveals additional genetic associations that are not apparent in single-phenotype GWAS in the same cohort.

All subtypes are consistent with known demographic, environmental, and disease relationships based on self-reported information, supporting their biological relevance. However, replication based on gold standard doctor diagnoses and replication in independent populations is warranted to confidently confirm the proposed subtypes.

## Supplementary Information

Additional file 1: Text S1. Supplementary method details of data preprocessing, model definition and training, topic identification and extraction, GWAS and post-GWAS analysis, and validation in UK biobank. Table S1. Listing of analyzed variables. Table S2. Discretization points of continuous values to quantile bins. Table S3. Characteristics of participants included in the genome-wide association study (GWAS) analysis. Table S4. Quality control filters of the GWAS. Table S5. Linkage disequilibrium score of candidate cardiovascular-kidney-metabolic (CKM) syndrome subtypes. Table S6. Topic loading and prevalence of CKM syndrome subtypes in the population stratified by ethnicity. Table S7. Sensitivity analysis of CKM syndrome subtype prevalence as a function of cut-off threshold. Tables S8 & S9. GWAS results without adjusting for factors related to obesity (body mass index, waist-to-hip ratio, and weight) as covariates in the GWAS model. Table S10. Age distribution per group of anthropometric assessments. Tables S11, S12 & S13. Age distribution per blood and urine biomarker level. Table S14. Sequencing depth of epigenetic markers across six organs. Table S15. Disease definitions of participants from UK biobank based on hospital admission records and death diagnoses. Figure S1. Generalization performance as a function of the number of topics. Figure S2. Convergence metrics of the Markov chain Monte Carlo simulations. Figure S3. Replication of topics in different Markov chains. Figure S4. Quantile-quantile plots of GWAS results per genotype chip (before meta analysis). Figure S5. Manhattan plots of GWAS results per genotype chip (before meta analysis). Figure S6. Topic loading of unselected subset of patients. Figure S7. Quantile-quantile and Manhattan plot of GWAS without BMI, waist-to-hip ratio and weight as covariates in the GWAS model. Figures S8 & S9. Tissue specificity of GWAS hits per CKM subtype, quantified by H3K27Ac histone modification. Figure S10. Over representation analysis of nearest genes identified in GWAS, with the op 20 hits in KEGG and GO databases. Figure S11. Association between candidate CKM subtype polygenic risk scores with cardiovascular disease, kidney disease, and type 2 diabetes in UK biobank.

## Data Availability

Access to research data of the Lifelines biobank was licensed from the company Lifelines (project ID ov21_00208) and the authors are under the contractual obligation that prevents us from sharing the data in any way. External validation was based on third party data from UK biobank (application number 206855), and the authors do not have permission to distribute the data. Visit https://www.ukbiobank.ac.uk/use-our-data/apply-for-access/ for details on how data may be accessed for research.

## Abbreviations

ALB: Albumin
ALP: Alkaline phosphatase
ALT: Alanine aminotransferase
APOA1: Apolipoprotein a1
AST: Aspartate aminotransferase
BMI: Body mass index
CHOL: Total cholesterol
Chr: Chromosome
CKM: Cardiovascular-kidney-metabolic
CREA: Creatinine
CRP: C-reactive protein
CVD: Cardiovascular disease
EA: Effect allele
FIB-4: Fibrosis-4 index
GC: Genomic control
GGT: $$\gamma$$-glutamyltransferase
GLU: Glucose
GWAS: Genome-wide association study
HBA1C: Glycated haemoglobin
HDL: High-density lipoprotein cholesterol
HGVS: Human Genome Variation Society nomenclature
LDL: Low-density lipoprotein cholesterol
MAF: Minor allele frequency
NEA: Non-effect allele
Pos: Genomic position on the human reference genome GRCh37
QQ: Quantile-quantile
rsID: Reference single nucleotide variant cluster identifier
SE: Standard error
SNV: Single nucleotide variant
TRIG: Triglyceride
T2DM: Type 2 diabetes mellitus
UA: Uric acid
WHR: Waist-to-hip ratio

## Footnotes

1 Pre-diabetes: 39–47 mmol/mol HbA1c [22].

2 Hyperglycaemia: $\ge 48$ mmol/mol HbA1c [22].

3 Desirable total cholesterol: $<5.17$ mM [23].

4 Optimal or near optimal levels of LDL: $\le 3.34$ mM [23].

5 Borderline high LDL cholesterol: 5.17−6.18 mM [23].

6 Border line high total cholesterol: 3.35−4.11 mM [23].

7 High LDL cholesterol: $\ge 4.90$ mM [23]

8 High total cholesterol: $>6.18$ mM [23].

9 Dyslipidemia: defined as hypercholesterolemia ($>5$ mM LDL cholesterol [25]), hypertriglyceridemia ($\ge 1.52$ mM triglycerides [1]), or combined dyslipidemia (mild-to-moderate elevated cholesterol and triglyceride levels [26]) [25].

## References

[1] Ndumele CE, Rangaswami J, Chow SL, Neeland IJ, Tuttle KR, Khan SS, et al. Cardiovascular-kidney-metabolic health: a presidential advisory from the American Heart Association. Circulation. 2023;148(20):1606–35. 37807924 10.1161/CIR.0000000000001184

[2] Manabe I. Chronic inflammation links cardiovascular, metabolic and renal diseases. Circ J. 2011;75(12):2739–48. 22067929 10.1253/circj.cj-11-1184

[3] Kadowaki T, Maegawa H, Watada H, Yabe D, Node K, Murohara T, et al. Interconnection between cardiovascular, renal and metabolic disorders: a narrative review with a focus on Japan. Diabetes Obes Metab. 2022;24(12):2283–96. 35929483 10.1111/dom.14829 PMC9804928

[4] Marassi M, Fadini GP. The cardio-renal-metabolic connection: a review of the evidence. Cardiovasc Diabetol. 2023;22(1):195. 37525273 10.1186/s12933-023-01937-x PMC10391899

[5] Sebastian SA, Padda I. Johal G. Cardiovascular-kidney-metabolic (CKM) syndrome: a state-of-the-art review. Curr Probl Cardiol. 2024;49(2):102344. 10.1016/j.cpcardiol.2023.102344 38103820

[6] Al-Chalabi S, Syed AA, Kalra PA, Sinha S. Mechanistic links between central obesity and cardiorenal metabolic diseases. Cardiorenal Med. 2024;14(1):12–22. 38171343 10.1159/000535772

[7] Aggarwal R, Ostrominski JW, Vaduganathan M. Prevalence of Cardiovascular-Kidney-Metabolic syndrome stages in US adults. JAMA. 2024;331(21):1858–60. 38717747 10.1001/jama.2024.6892 PMC11079779

[8] Li J, Lu L, Zhang Y, Gu Q, Zhu T, Jing F, et al. Regional rrevalence, stage distribution, and temporal trends of cardiovascular-kidney-metabolic syndrome in the Americas, Europe and Western Pacific: A Systematic Review and Meta-Analysis. medRxiv. 2025. 10.1101/2025.06.04.25328945.

[9] Khan SS, Coresh J, Pencina MJ, Ndumele CE, Rangaswami J, Chow SL, et al. Novel prediction equations for absolute risk assessment of total cardiovascular disease incorporating cardiovascular-kidney-metabolic health: a scientific statement from the American Heart Association. Circulation. 2023;148(24):1982–2004. 37947094 10.1161/CIR.0000000000001191

[10] Ndumele CE, Neeland IJ, Tuttle KR, Chow SL, Mathew RO, Khan SS, et al. A synopsis of the evidence for the science and clinical management of cardiovascular-kidney-metabolic (CKM) syndrome: a scientific statement from the American Heart Association. Circulation. 2023;148(20):1636–64. 37807920 10.1161/CIR.0000000000001186

[11] Zeng Z, Xiao Z. Cardiometabolic risk phenotypes and chronic kidney disease incidence in older adults: a nationwide longitudinal cohort study. BMC Public Health. 2025;25(1):2581. 40730993 10.1186/s12889-025-23868-w PMC12309190

[12] McCoy TH, Castro VM, Snapper LA, Hart KL, Perlis RH. Efficient genome-wide association in biobanks using topic modeling identifies multiple novel disease loci. Mol Med. 2017;23:285–94. 28861588 10.2119/molmed.2017.00100 PMC5681697

[13] Zhang Y, Jiang X, Mentzer AJ, McVean G, Lunter G. Topic modeling identifies novel genetic loci associated with multimorbidities in UK Biobank. Cell Genom. 2023;3(8):100371. 37601973 10.1016/j.xgen.2023.100371 PMC10435382

[14] Scholtens S, Smidt N, Swertz MA, Bakker SJ, Dotinga A, Vonk JM, et al. Cohort Profile: LifeLines, a three-generation cohort study and biobank. Int J Epidemiol. 2015;44(4):1172–80. 25502107 10.1093/ije/dyu229

[15] Chaudhary K, Malhotra K, Sowers J, Aroor A. Uric acid-key ingredient in the recipe for cardiorenal metabolic syndrome. Cardiorenal Med. 2013;3(3):208–20. 24454316 10.1159/000355405 PMC3884201

[16] Bhale AS, Venkataraman K. Leveraging knowledge of HDLs major protein ApoA1: structure, function, mutations, and potential therapeutics. Biomed Pharmacother. 2022;154:113634. 36063649 10.1016/j.biopha.2022.113634

[17] Ferrannini G, Rosenthal N, Hansen MK, Ferrannini E. Liver function markers predict cardiovascular and renal outcomes in the CANVAS program. Cardiovasc Diabetol. 2022;21(1):127. 35787704 10.1186/s12933-022-01558-w PMC9254689

[18] ElSayed NA, Aleppo G, Aroda VR, Bannuru RR, Brown FM, Bruemmer D, et al. 11. Chronic Kidney Disease and Risk Management: Standards of Care in Diabetes—2023. Diabetes Care. 2022;46(Supplement_1):S191–202. 10.2337/dc23-S011 PMC9810467 36507634

[19] Erosheva E, Fienberg S, Lafferty J. Mixed-membership models of scientific publications. Proc Natl Acad Sci. 2004;101(suppl_1):5220–5227. 10.1073/pnas.0307760101 PMC387299 15020766

[20] Lu HM, Wei CP, Hsiao FY. Modeling healthcare data using multiple-channel latent Dirichlet allocation. J Biomed Inform. 2016;60:210–23. 26898516 10.1016/j.jbi.2016.02.003

[21] Neijzen D, Donker HC, Vonk J, Lunter G. Unsupervised Learning in Heterogeneous Tabular Data: Application to a Respiratory Disease Cohort. 2025. Available at SSRN 5372663. 10.2139/ssrn.5372663.

[22] Association AD. 2. Classification and Diagnosis of Diabetes: Standards of Medical Care in Diabetes—2021. Diabetes Care. 2020;44(Supplement_1):S15–33. 10.2337/dc21-S002. 10.2337/dc21-S002 33298413

[23] Cleeman JI. Executive summary of the third report of the National Cholesterol Education Program (NCEP) expert panel on detection, evaluation, and treatment of high blood cholesterol in adults (Adult Treatment Panel III). JAMA. 2001;285(19):2486–97. 10.1001/jama.285.19.2486 11368702

[24] Kramer HJ, Jaar BG, Choi MJ, Palevsky PM, Vassalotti JA, Rocco MV. An endorsement of the removal of race from GFR estimation equations: a position statement from the National Kidney Foundation Kidney Disease Outcomes Quality Initiative. Am J Kidney Dis. 2022;80(6):691–6. 36058427 10.1053/j.ajkd.2022.08.004

[25] Berberich AJ, Hegele RA. A modern approach to dyslipidemia. Endocr Rev. 2022;43(4):611–53. 34676866 10.1210/endrev/bnab037 PMC9277652

[26] Gill PK, Dron JS, Berberich AJ, Wang J, McIntyre AD, Cao H, et al. Combined hyperlipidemia is genetically similar to isolated hypertriglyceridemia. J Clin Lipidol. 2021;15(1):79–87. 33303402 10.1016/j.jacl.2020.11.006

[27] Coughlin SR, Mawdsley L, Mugarza JA, Calverley PM, Wilding JP. Obstructive sleep apnoea is independently associated with an increased prevalence of metabolic syndrome. Eur Heart J. 2004;25(9):735–41. 15120883 10.1016/j.ehj.2004.02.021

[28] Cappuccio FP, Miller MA. Sleep and cardio-metabolic disease. Curr Cardiol Rep. 2017;19:1–9. 28929340 10.1007/s11886-017-0916-0 PMC5605599

[29] Huang X, Liang J, Zhang J, Fu J, Xie W, Zheng F. Association of cardiovascular-kidney-metabolic health and social connection with the risk of depression and anxiety. Psychol Med. 2024;54(15):4203-11. 39552398 10.1017/S0033291724002381 PMC11650165

[30] Buniello A, MacArthur JAL, Cerezo M, Harris LW, Hayhurst J, Malangone C, et al. The NHGRI-EBI GWAS Catalog of published genome-wide association studies, targeted arrays and summary statistics 2019. Nucleic Acids Res. 2019;47(D1):D1005–12. 30445434 10.1093/nar/gky1120 PMC6323933

[31] Marioni RE, Harris SE, Zhang Q, McRae AF, Hagenaars SP, Hill WD, et al. GWAS on family history of Alzheimer’s disease. Transl Psychiatry. 2018;8(1):99. 29777097 10.1038/s41398-018-0150-6 PMC5959890

[32] Wang SH, Su MH, Chen CY, Lin YF, Feng YCA, Hsiao PC, et al. Causality of abdominal obesity on cognition: a trans-ethnic Mendelian randomization study. Int J Obes. 2022;46(8):1487–92. 10.1038/s41366-022-01138-8 35538205

[33] Farruggia MC, Small DM. Effects of adiposity and metabolic dysfunction on cognition: a review. Physiol Behav. 2019;208:112578. 31194997 10.1016/j.physbeh.2019.112578 PMC6625347

[34] Lorenzini PA, Langley SR. Multi-omics and Mendelian randomization network analysis of the association between metabolic and cognitive functions in the UK Biobank database. medRxiv. 2024;2024–05. 10.1101/2024.05.30.24307732.

[35] Herrera BM, Lindgren CM. The genetics of obesity. Curr Diab Rep. 2010;10:498–505. 20931363 10.1007/s11892-010-0153-z PMC2955913

[36] Kirchler M, Konigorski S, Norden M, Meltendorf C, Kloft M, Schurmann C, et al. transferGWAS: GWAS of images using deep transfer learning. Bioinformatics. 2022;38(14):3621–8. 35640976 10.1093/bioinformatics/btac369

[37] Radhakrishnan A, Friedman SF, Khurshid S, Ng K, Batra P, Lubitz SA, et al. Cross-modal autoencoder framework learns holistic representations of cardiovascular state. Nat Commun. 2023;14(1):2436. 37105979 10.1038/s41467-023-38125-0 PMC10140057

[38] Yun T, Cosentino J, Behsaz B, McCaw ZR, Hill D, Luben R, et al. Unsupervised representation learning on high-dimensional clinical data improves genomic discovery and prediction. Nat Genet. 2024;56(8):1604–13. 38977853 10.1038/s41588-024-01831-6 PMC11319202

[39] Xie Z, Zhang T, Kim S, Lu J, Zhang W, Lin CH, et al. IgwAS: image-based genome-wide association of self-supervised deep phenotyping of retina fundus images. PLoS Genet. 2024;20(5):e1011273. 38728357 10.1371/journal.pgen.1011273 PMC11111076

[40] Higashiyama A, Murakami Y, Hozawa A, Okamura T, Hayakawa T, Kadowaki T, et al. Does self-reported history of hypertension predict cardiovascular death? Comparison with blood pressure measurement in a 19-year prospective study. J Hypertens. 2007;25(5):959–64. 17414658 10.1097/HJH.0b013e3280586735

[41] Sluijs I, van der A D, Beulens J, Spijkerman A, Ros M, Grobbee D, et al. Ascertainment and verification of diabetes in the EPIC-NL study. Neth J Med. 2010;68(1):333–9. 20739736

[42] Goto A, Morita A, Goto M, Sasaki S, Miyachi M, Aiba N, et al. Validity of diabetes self-reports in the Saku diabetes study. J Epidemiol. 2013;23(4):295–300. 10.2188/jea.JE20120221 PMC3709549 23774288

[43] Carpio EM, Ashworth M, Asgari E, Shaw C, Schartau P, Durbaba S, et al. Hypertension and cardiovascular risk factor management in a multi-ethnic cohort of adults with CKD: a cross sectional study in general practice. J Nephrol. 2022;35(3):901–10. 10.1007/s40620-021-01149-0 PMC8995266 34782969

[44] Klijs B, Scholtens S, Mandemakers JJ, Snieder H, Stolk RP, Smidt N. Representativeness of the LifeLines cohort study. PLoS One. 2015;10(9):e0137203. 26333164 10.1371/journal.pone.0137203 PMC4557968

