# Supplementary material for: Cardiovascular-kidney-metabolic syndrome: candidate subtypes and genetic risk factors
Source: BMC Med Genomics. 2026 Jan 31;19:47. doi: 10.1186/s12920-026-02315-8 (PMC13014838; doi:10.1186/s12920-026-02315-8)
Supplement: Supplementary file 1 — Additional file 1: Text S1. Supplementary method details of data preprocessing, model definition and training, topic identification and extraction, GWAS and post-GWAS analysis, and validation in UK biobank. Table S1. Listing of analyzed variables. Table S2. Discretization points of continuous values to quantile bins. Table S3. Characteristics of participants included in the genome-wide association study (GWAS) analysis. Table S4. Quality control filters of the GWAS. Table S5. Linkage disequilibrium score of candidate cardiovascular-kidney-metabolic (CKM) syndrome subtypes. Table S6. Topic loading and prevalence of CKM syndrome subtypes in the population stratified by ethnicity. Table S7. Sensitivity analysis of CKM syndrome subtype prevalence as a function of cut-off threshold. Tables S8 & S9. GWAS results without adjusting for factors related to obesity (body mass index, waist-to-hip ratio, and weight) as covariates in the GWAS model. Table S10. Age distribution per group of anthropometric assessments. Tables S11, S12 & S13. Age distribution per blood and urine biomarker level. Table S14. Sequencing depth of epigenetic markers across six organs. Table S15. Disease definitions of participants from UK biobank based on hospital admission records and death diagnoses. Figure S1. Generalization performance as a function of the number of topics. Figure S2. Convergence metrics of the Markov chain Monte Carlo simulations. Figure S3. Replication of topics in different Markov chains. Figure S4. Quantile-quantile plots of GWAS results per genotype chip (before meta analysis). Figure S5. Manhattan plots of GWAS results per genotype chip (before meta analysis). Figure S6. Topic loading of unselected subset of patients. Figure S7. Quantile-quantile and Manhattan plot of GWAS without BMI, waist-to-hip ratio and weight as covariates in the GWAS model. Figures S8 & S9. Tissue specificity of GWAS hits per CKM subtype, quantified by H3K27Ac histone modification. Figure S10. Over repre [file 12920_2026_2315_MOESM1_ESM.pdf]

Supplemental Materials for Cardio-Kidney-Metabolic Syndrome: Candidate Subtypes and Genetic Risk Factors

S1 Supplementary Methods

S1.1 Feature engineering and definitions used in Lifelines dataset

Table S1 List of variables of the Lifelines biobank used in the analysis. All measurements correspond to the first assessment.

Demographics	Anthropometrics	Blood measurements	Spot urine measurements	Questionnaire (self-reported)	Diagnosis
Age	Hip circumference	Alanine Aminotransferase	Albumin	Agoraphonia	Cardiovascular disease
Ethnicity	Length	Alkaline Phosphatase	Creatinine	Anxiety	
Gender	Mass	Apolipoprotein A1		Attention deficit hyperactivity disorder	
	Waist circumference	Aspartate Aminotransferase		Atherosclerosis	
		C-reactive protein		Bipolar disorder	
		Creatinine		Burnout	
		γ-Glutamyltransferase		Depression	
		Glucose		Hypertension	
		High Density Lipoprotein		Kidney disease	
		Low Density Lipoprotein		Liver Cirrhosis	
		Pressure (sys- and diastolic)		Obsessive–compulsive disorder	
		Thrombocytes		Panic disorder	
		Total cholesterol		Schizophrenia	
		Triglyceride		Sleep	
		Urea		Social phobia	
		Uric Acid		Type-II diabetes mellitus	

Demographics, anthropometrics, blood measurements, spot urine measurements, and questionnaire data from the baseline assessment were obtained from the Lifelines biobank [1] (Table S1). In addition, a curated cardio vascular disease definition from Ref. [2] was used based on data of [3]. Based on these measurements, the liver fibrosis index FIB-4 (as described in Ref. [4]), body mass index (BMI) and the waist-to-hip ratio (WHR) were calculated. These measurements were included because of their known involvement in heart disease, kidney disease and type-II diabetes mellitus [5–11]. BMI was discretized according to the classification of the world health organization [12]. Since mental health disorders are a known risk factor of CKM [11], we denote the presence of a mental health disorder when a participant indicated the presence of any of the following conditions: agoraphonia, attention deficit hyperactivity disorder, anxiety, bipolar disorder, burnout, depression, obsessive–compulsive disorder, panic disorder, schizophrenia, and social phobia. Sleep is categorized according to health risk as < 6 hours (short), 6–8 hours, and > 8 hours (long) [13]. Follow-up questionnaire data on kidney disorder, type-II diabetes, heart attack, heart failure and chest pain were obtained from assessments 2a, 3a and 3b. The latter three variables were used to define self-reported cardio vascular disease, when the participant indicated heart attack or failure or marked the question "had chest pain, or other complaints such as a burdensome, oppressive or heavy feeling on the chest" as either "somewhat", "quite a lot", or "very much". Age, blood and urine measurements (except for blood pressure and thrombocytes) and the derived quantities, were quantile transformed stratified by sex and discretized into five percentile buckets: [0–10%, 10–30%,30–70%,70–90%,90–100%] (Table S2). This choice was a compromise between resolution at the tails and not having too many features. Blood pressure was classified as normal, elevated, or hypertension according to the guidelines of the European

Table S2 Discretization of continuous values in percentile buckets, stratified by sex. All biomarkers refer to concentrations in blood, unless specified otherwise.

	**Percentile**	**0%**	**10%**	**30%**	**70%**	**90%**	**100%**
	**Sex**						
Age (years)	female	18	28	40	51	63	92
	male	19	30	41	53	65	88
Waist-hip-ratio (-)	female	0.62	0.77	0.83	0.91	0.96	1.28
	male	0.70	0.88	0.93	1.00	1.05	1.53
Mass (kg)	female	40	59	66	79	92	155
	male	52	73	81	93	105	184
Alanine aminotransferase (U/l)	female	1	10	13	20	28	295
	male	2	16	21	33	48	195
Aspartate aminotransferase (U/l)	female	11	16	19	23	28	158
	male	9	19	23	29	36	114
Alkaline Phosphatase (U/l)	female	19	40	49	66	81	323
	male	1	46	56	71	85	348
γ-Glutamyltransferase (U/l)	female	4	11	14	21	34	516
	male	7	16	21	35	58	1287
High-sensitivity C-reactive protein (mg/l)	female	0.20	0.40	0.80	2.80	7.40	86.20
	male	0.20	0.30	0.60	1.80	4.40	247.00
Total cholesterol (mM)	female	2.10	3.80	4.50	5.50	6.40	9.90
	male	2.00	3.90	4.60	5.60	6.40	18.60
High density lipoprotein cholesterol (mM)	female	0.40	1.19	1.40	1.80	2.20	3.70
	male	0.40	1.00	1.10	1.40	1.70	3.00
Low density lipoprotein cholesterol (mM)	female	0.40	2.09	2.60	3.50	4.30	7.60
	male	0.60	2.20	2.90	3.80	4.50	7.70
Creatinine (μM)	female	38	56	62	71	79	324
	male	17	70	77	87	96	581
Glucose (mM)	female	2.70	4.30	4.60	5.00	5.50	14.90
	male	2.90	4.50	4.80	5.30	5.80	22.10
Glycated haemoglobin (mmol/mol)	female	16.00	32.91	35.00	39.00	42.00	95.00
	male	19.00	33.00	35.00	39.00	42.00	127.00
Apolipoprotein A1 (g/l)	female	0.78	1.27	1.45	1.71	1.94	2.74
	male	0.56	1.14	1.29	1.51	1.70	2.47
Triglyceride (mM)	female	0.17	0.52	0.71	1.13	1.65	9.89
	male	0.20	0.62	0.88	1.52	2.37	28.42
Uric acid (mM)	female	100	190	230	280	330	600
	male	130	260	310	370	421	650
Urea (mM)	female	1.80	3.50	4.20	5.30	6.30	11.70
	male	1.70	4.10	4.90	6.20	7.20	32.90
Fibrosis-4 index (years $\cdot$ $U^{0.5} \cdot l^{0.5}$)	female	0.22	0.51	0.71	1.08	1.46	4.01
	male	0.23	0.58	0.79	1.22	1.71	4.97
Urine albumin (mg/l)	female	0.00	0.60	1.30	3.20	7.21	394
	male	0.00	0.70	1.50	3.70	8.20	5896
Urine creatinine (mM)	female	1.60	4.90	7.10	12.5	17.9	45.9
	male	2.4	7.2	10.5	16.7	22.0	42.6

Society of Cardiology [14]. Finally, discretized features were encoded one-hot, where missing values are represented as all zeros.

S1.2 Model

Each one-hot encoded blood and urine measurements was modelled using a separate multinomial draw from a generalization of latent Dirichlet allocation [15–17]. Briefly, the dataset comprises $i = 1, \ldots, m$ training examples of $j = 1, \ldots, c$ multinomial channels each, with n_j features per channel j. In other words, the dataset is made of c multinomially distributed tabular blocks $\boldsymbol{x}_j \in \mathbb{N}_0^{(m \times n_j)}$. It is assumed that the data is explained by $k = 1, \ldots, K$ latent components (i.e., profiles/topics) according to the generative model:

- Sample hidden units $\boldsymbol{h}^{(i)} \sim \mathrm{Dir}(\boldsymbol{\alpha})$, for $i = 1 \ldots m$
- Sample weights $\boldsymbol{w}_{jk} \sim \mathrm{Dir}(\boldsymbol{\beta}_{jk})$, $k = 1 \ldots K, j = 1 \ldots c$
- For multinomial $j = 1 \ldots c$ and item $t = 1 \ldots x_j^{(i)}$:
  - Choose a component $k = z_{jt}^{(i)} \sim \mathrm{Cat}(\boldsymbol{h}^{(i)})$
  - Draw an item $\chi_{jt}^{(i)} \sim \mathrm{Cat}(\boldsymbol{w}_{jk})$

where $\boldsymbol{\alpha}$ and $\boldsymbol{\beta}_{jk}$ are the hyperparameters of the hidden units $\boldsymbol{h}^{(i)}$ and weights $\boldsymbol{w}_{jk}$, respectively. Henceforth we will work in the multinomial representation $x_{jl}^{(i)} = \sum_{t=1}^{x_j^{(i)}} \delta(\chi_{jt}^{(i)}, l)$ and write $\boldsymbol{X} = \left[\boldsymbol{x}^{(1)}, \ldots, \boldsymbol{x}^{(m)}\right]^T$. We have used the notation that a scalar $x_j^{(i)} = \sum_{l=1}^{n_j} x_{jl}^{(i)}$ is the marginal sum of the vector. The model was trained by collapsed Gibbs sampling the latent components $z_{jt}^{(i)}$ after integrating out the hidden units and weights. Briefly, for each sequence element t in channel j of training example i, sample the topic assignment $z_{jt}^{(i)}$ according to

$$p(z_{jt}^{(i)}{=}k, \chi_{jt}^{(i)}{=}l | \boldsymbol{X} - \chi_{jt}^{(i)}, \boldsymbol{Z} - z_{jt}^{(i)}) = \frac{(\alpha_k{+}y_k^{(i)})(\beta_{jkl}{+}y_{jkl})}{(\alpha + y^{(i)})(\beta_{jk} + y_{jk})}, \tag{S1}$$

where y are the summary statistics of the sequence elements with element $(\chi_{jt}^{(i)}, z_{jt}^{(i)})$ removed:

$$y_{j'kl}^{(i')} = \sum_{\tau=1}^{x_{j'}^{(i')}} \delta(\chi_{j'\tau}^{(i')}, l)\delta(z_{j'\tau}^{(i')}, k) - \delta(i, i')\delta(j, j')\delta(\chi_{jt}^{(i)}, l)\delta(z_{jt}^{(i)}, k), \tag{S2}$$

where we used the shorthand $y_{jk} = \sum_{l=1}^{n_j} y_{jkl} = \sum_{l=1}^{n_j} \sum_{i=1}^{m} y_{jkl}^{(i)}$. A single step in the Markov chain Monte Carlo simulation is completed when all individual topic assignments $z_{jt}^{(i)}$ have been sampled. Making use of the Dirichlet-multinomial conjugacy relation, hidden units are then sampled $\boldsymbol{h}^{(i)} \sim \mathrm{Dir}(\boldsymbol{\alpha}{+}\boldsymbol{y}^{(i)})$ and weights were sampled $\boldsymbol{w}_{jk} \sim \mathrm{Dir}(\boldsymbol{\beta}_{jk}{+}\boldsymbol{y}_{jk})$, where the summary statistics $\boldsymbol{y}$ now include all sequence elements, including element t.

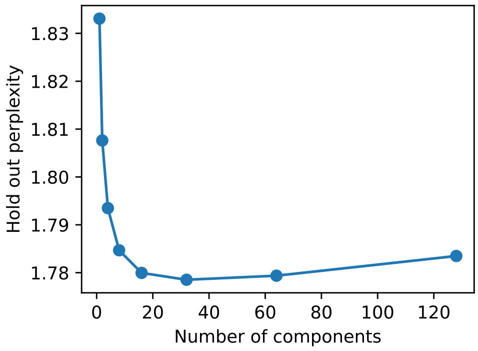

Fig. S1 Best generalization on unseen data (the development set) was observed with $K = 32$ topics. Lower perplexity, Eq. (S3), indicates better model performance.

S1.2.1 Hyperparameter tuning

Before training the full model, we first determined the best hyperparametes on a single Markov chain. The dataset was split into a training set $\boldsymbol{X}_{\text{train}}$ and development set $\boldsymbol{X}_{\text{dev}}$. Each subset contained the same set of m participants, but each multinomial observation, which is assumed to be independent and identically distributed given participant i, was split into 1:2 ratio between $\boldsymbol{X}_{\text{train}}$ and $\boldsymbol{X}_{\text{dev}}$ (so that the original dataset is $\boldsymbol{X}_{\text{train}} + \boldsymbol{X}_{\text{dev}}$). We varied the number of components, $K = 2^0, 2^1, \ldots, 2^7$, trained the model for 256 burn in steps on $\boldsymbol{X}_{\text{train}}$ and collected $S = 256$ subsequent samples from the Markov chain to estimate the posterior average of the hidden units $\overline{\boldsymbol{H}} = S^{-1} \sum_{\sigma=1}^{S} \boldsymbol{H}^{(\sigma)}$ and weights $\overline{\boldsymbol{W}} = S^{-1} \sum_{\sigma=1}^{S} \boldsymbol{W}^{(\sigma)}$ where the superscript (σ) denotes a sample σ collected from the Markov chain. Performance was quantified in terms of perplexity $\mathcal{L}(\mathbf{X}^{\text{dev}})$ on unseen observations from the development set

$$\mathcal{L}(\mathbf{X}) = \exp\left(\frac{-1}{mc} \sum_{i=1}^{m} \sum_{j=1}^{c} \frac{\sum_{l=1}^{n_j} x_{jl}^{(i)} \ln p_{jl}^{(i)}}{x_j^{(i)}} \right), \tag{S3}$$

where $p_{jl}^{(i)} = \sum_{k=1}^{K} \overline{h}_k^{(i)} \overline{w}_{jkl}$. Lower perplexity is better and measures how well the model can compress the data compared to an uninformative (i.e., uniform) model. With initial hyperparameters $\boldsymbol{\alpha} = 1/K$ and $\boldsymbol{\beta} = 1$ we found $K = 32$ gave the best generalization (Fig. S1). Holding $K = 32$ fixed, a small grid search over $\boldsymbol{\alpha} = \frac{2^{-2}}{K}, \ldots, \frac{2^2}{K}$ and $\boldsymbol{\beta} = 2^{-2}, \ldots, 2^2$ revealed that $\boldsymbol{\alpha} = \frac{1}{2K}$ and $\beta = 1$ gave the best perplexity on the dev set.

S1.3 Model training

With these hyperparameters, eight separate Markov chains were run on the full dataset (i.e., $\boldsymbol{X}_{\text{train}} + \boldsymbol{X}_{\text{dev}}$) for 38,400 Gibbs steps thinning to every 200th sample. In terms of likelihood, all chains converged after a few hundred steps (Fig. S2a). We conservatively discarded the first 64 thinned samples of each chain (corresponding to a

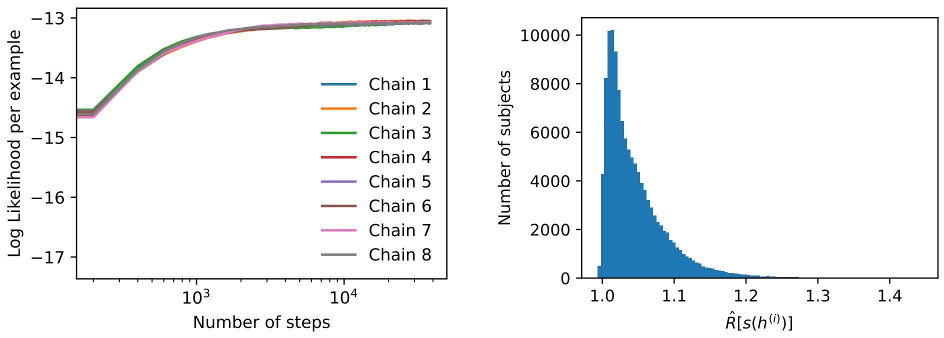

a

b

Fig. S2 The Markov chain Monte Carlo simulations have converged and mixed. (a) The log likelihood per training example (i.e., $L(\boldsymbol{X}) = m^{-1}\sum_{i=1}^{m}\left(\sum_{j=1}^{c}\ln\frac{x_j^{(i)}!}{\prod_{l'} x_{jl}^{(i)}!} + \sum_{l=1}^{n_j} x_{jl}^{(i)}\ln[\sum_{k=1}^{K} h_k^{(i)} w_{jkl}]\right)$) as a function of the number of Gibbs steps, for independent Markov chains. (b) For each participant i, between- and within-chain estimates of the entropy of the hidden units $s(\boldsymbol{h}^{(i)}) = -\sum_{k=1}^{K} h_k^{(i)} \ln h_k^{(i)}$ are evaluated using the $\hat{R}$ convergence diagnostic. The figure shows the distribution over the values i (training examples).

burn-in of 12,800 Gibbs steps) so that we had $8 \times 128 = 8192$ samples in total. As a measure of mixing, we evaluated for each participant, i, the entropy of the hidden units $s^{(i)} = -\sum_{k=1}^{K} h_k^{(i)} \ln h_k^{(i)}$ (which is invariant under permutations of the components) and compared variance between- and within chain estimates using $\hat{R}$ [18], where $\hat{R} \leq 1.1$ indicates good mixing between chains. The Markov chains showed excellent convergence for essentially all participants (Fig. S2b).

S1.4 Topic identification

To solve the topic identifiability problem, we matched topics of different chains to its' centroid by repeatedly solving the optimal transport problem [19] using the Hungarian algorithm [20] until the centroid converged in terms of silhouette score [21]. Distance between topics was measured by constructing one matrix per topic by concatenating all channels. These matrices are then used to compute the cosine distance. The centroid was initialised with restarting points coming from different chains and the consensus topic that gave the best silhouette score were selected. Three out of 32 topics had a poor silhouette score, because they had overlap with other topics (Fig. S3). These were excluded for further analysis. Demographics (discretized age, ethinicity, sex, smoking status), anthropometrics (body mass index classes, and discretized waist-hip-ratio and weight), and life style (discretized sleeping hours) were associated by multivariate regression analysis on rank-based inverse normal transformation on the posterior average of the hidden units, with a 29-fold Bonferroni multiple testing correction. The same approach was taken for association with self-reported disease outcomes, with a separate multivariate regression per time point (baseline, second assessment 2a, third assessment 3a, and first follow-up questionnaire of the second assessment 3b).

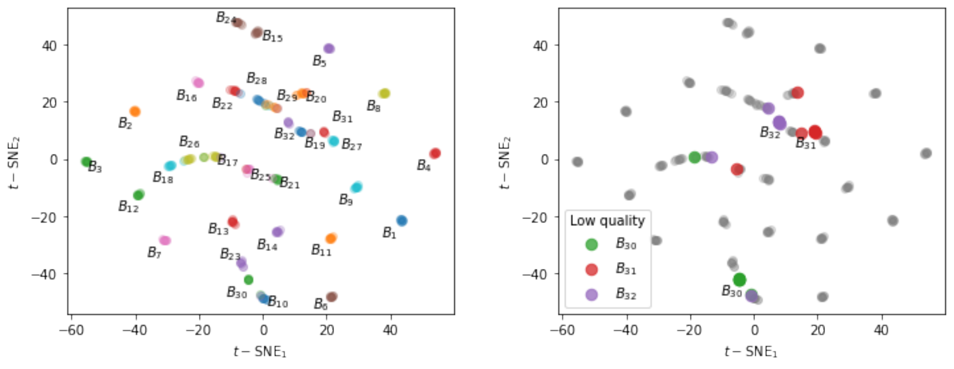

a b

Fig. S3 Nearly all topics are accurately recapitulated in eight independent Markov chains. (a) Clustering of eight independent Markov chain Monte Carlo simulations (coloured by topic) using a t-distributed stochastic neighbour embedding. (b) Three low quality topics, where different Markov chains don't cluster, but overlap with other topics have been excluded. Embeddings were generated using sci-kit learn with perplexity set to 8, and otherwise default settings.

S1.5 Genome wide association study in Lifelines

Lifelines participants were genotyped with three sets of microarrays.

- Illumina Infinium HumanCytoSNP-12v2 (CytoSNP, 15,422 samples). Genotypes were imputed against the 1000 genomes panel and the GoNL [22] panel (containing genomes from Dutch ancestry) using IMPUTE2 [23].
- Illumina Infimium Global Screening Array MultiEthnic Disease Version (GSA, 36,339 samples). A final set of 571,420 quality controlled autosomal and X chromosome markers were used to impute genotypes using the Sanger imputation service from the Haplotype Reference Consortium panel [24].
- FinnGen Thermo Fisher Axiom® custom array (Affymetrix, 28,284 samples). A total of 462,731 markers (from autosomal and X chromosomes) passing quality control checks were used to impute samples using the same approach as with the GSA microarray.

All imputed markers refer to human reference genome build GRCH37.

Some individuals were genotyped more than once. We removed duplicate genotypes and 1st degree relatives from the lower quality CytoSNP microarray. From the remaining samples with overlap or 1st degree relatives in the GSA and Affymetrix microarray, we prioritized samples from the GSA microarray. We analyzed autosomal chromosomes only, and excluded subjects without sex information. To avoid false positive hits due to population structure, we excluded non-white or unknown ethnicity and removed samples where either of the first two principal components exceeded five standard deviations from the average. Each microarray subcohort was analyzed separately to prevent batch effects. Next, we used PLINK [25] to quality control the imputed genotype data (Table. S4) and used Regenie [26] to fit the weights $\boldsymbol{w}$ of a

Table S3 Characteristics of genotyped participants included in GWAS analysis. Mean: μ; standard deviation: σ.

Genotype chip		Affymetrix	CytoSNP	GSA
m		23,956	6,381	22,657
age (years), $\mu\,(\sigma)$		45.0 (13.5)	48.6 (10.7)	44.3 (14.3)
sex, m (%)	female	15,120 (63.1)	3,710 (58.1)	13,487 (59.5)
	male	8,836 (36.9)	2,671 (41.9)	9,170 (40.5)
smoker, m (%)	current-recent	4,835 (20.2)	1,512 (23.7)	4,132 (18.2)
	ex	7,840 (32.7)	2,325 (36.4)	7,383 (32.6)
	never	11,281 (47.1)	2,544 (39.9)	11,142 (49.2)
Body mass index, m (%)	normal	11,184 (46.7)	2,731 (42.8)	10,970 (48.4)
	obesity-CI	2,693 (11.2)	758 (11.9)	2,259 (10.0)
	obesity-CII	635 (2.7)	201 (3.1)	496 (2.2)
	obesity-CIII	209 (0.9)	58 (0.9)	165 (0.7)
	overweight	9,027 (37.7)	2,593 (40.6)	8,559 (37.8)
	underweight	208 (0.9)	40 (0.6)	208 (0.9)
waist-hip ratio (–), $\mu\,(\sigma)$		0.9 (0.1)	0.9 (0.1)	0.9 (0.1)
weight (kg), $\mu\,(\sigma)$		79.0 (15.0)	80.2 (15.3)	78.8 (14.7)

Table S4 PLINK2 settings used to quality control the genotype data.

**Genotype chip**	`--geno`	`--hwe`	`--mac`	`--maf`	`--mind`	`--indep-pairwise`
CytoSNP	10^{-1}	10^{-15}	10^{2}	$5 \cdot 10^{-2}$	10^{-1}	10^{3}, 10^{2}, 0.9
GSA	10^{-1}	10^{-15}	10^{2}	10^{-2}	10^{-1}	10^{3}, 10^{2}, 0.9
Affymetrix	10^{-1}	10^{-15}	10^{2}	10^{-2}	10^{-1}	10^{3}, 10^{2}, 0.9

Table S5 Intercept of linkage disequilibrium score regression on summary statistics of genome-wide association study with both demographic features (sex, age, smoking status) and anthropometrics (weight, waist-hip-ratio, and body mass index). Estimates and standard error are listed per blood-urine profile as a quantitative trait (rows), and per genotyping platform (columns).

Quantitative trait	Intercept (standard error)		
	Affymetrix	CytoSNP	GSA
B_2	1.0148 (0.0069)	0.9999 (0.0082)	0.9867 (0.0067)
B_3	1.0051 (0.0063)	0.9968 (0.0071)	0.9999 (0.0066)
B_{16}	1.0128 (0.0068)	0.9861 (0.0074)	0.9846 (0.0068)
B_{22}	1.0264 (0.0071)	0.9965 (0.0074)	0.988 (0.0068)
B_{28}	1.0255 (0.0068)	0.9926 (0.0076)	0.9869 (0.0063)

Table S6 Per topic, distribution of posterior average $\bar{\mathbf{H}}$ in the population, stratified by self-reported ethnicity. Participants were considered subtype positive when the posterior average loading was more than 50%. Abbreviations: IQR, inter quartile range.

Subgroup		$\mathbf{B}_2$	$\mathbf{B}_3$	$\mathbf{B}_{16}$	$\mathbf{B}_{22}$	$\mathbf{B}_{28}$
**Loading [median (IQR)]**						
Ethnicity	Overall	0.25% (0.17–0.53)	0.32% (0.18–0.86)	0.26% (0.16–0.55)	0.30% (0.17–0.91)	0.40% (0.21–1.16)
	Asians	0.29% (0.17–0.72)	0.33% (0.18–0.93)	0.29% (0.17–0.63)	0.38% (0.17–1.64)	0.46% (0.25–1.25)
	Blacks	0.31% (0.18–1.14)	0.39% (0.19–0.85)	0.27% (0.17–0.59)	0.34% (0.17–1.09)	0.45% (0.20–1.64)
	Whites	0.25% (0.17–0.52)	0.32% (0.18–0.86)	0.26% (0.16–0.54)	0.30% (0.17–0.90)	0.40% (0.21–1.15)
	Other	0.27% (0.17–0.63)	0.32% (0.18–0.80)	0.26% (0.16–0.58)	0.32% (0.17–1.14)	0.42% (0.21–1.26)
**Subtype positive** n **(%)**						
Ethnicity	Overall	2,663 (2.18%)	2,286 (1.87%)	3,003 (2.46%)	1,843 (1.51%)	1,541 (1.26%)
	Asians	23 (3.85%)	13 (2.17%)	21 (3.51%)	16 (2.68%)	12 (2.01%)
	Blacks	8 (4.06%)	6 (3.05%)	4 (2.03%)	0 (0.00%)	4 (2.03%)
	Whites	2,561 (2.17%)	2,220 (1.88%)	2,887 (2.45%)	1,766 (1.50%)	1,473 (1.25%)
	Other	36 (2.23%)	21 (1.30%)	42 (2.60%)	33 (2.04%)	29 (1.79%)

linear model on $y = \boldsymbol{w} \cdot \boldsymbol{x} + b$. While obesity can be an explaining factor for an anomalous blood-urine profile, it can also be a mediator of a genetic variant. Therefor, two models were analyzed:

- model 1: adjusted for demographics (self-reported sex, age as a continuous variable, and smoking status) and ten principal components to account for population structure;
- model 2: which additionally included anthropometrics (body mass index, waist-hip-ratio, and weight; all continuous valued).

Inclusion of these variables were determined by forward selection with an inclusion threshold of $p = 0.1$. The posterior average of the labels y (the topic loadings as a quantitative trait), were rank-based inverse normal transformed before training to help make the residuals Gaussian. The minimum imputation quality was set to `--minINFO 0.7` and the minor allele count to `--minMAC 5`. After model training, rare variants (with a minor allele frequency $< 5\%$ for the CytoSNP chip with the smaller sample size, and $< 1\%$ for the two other larger datasets) were excluded, and we verified that the genomic inflation factor λ_{GC} for the remaining markers were close to one and that the QQ-plots showed no large trends of p-value inflation (Fig. S4). A linkage-disequilibrium score regression (using ldsc Python package [27] with European linkage disequilibrium scores from the 1000 genomes project) further confirmed the absence of confounding, with intercepts close to one (Table S5). Re-analysis of the data without BMI, WHR, and weight gave similar QQ-plots, inflation factors, and LD score regression intercepts (data not shown). Finally, result were combined by meta-analysis using metal [28] and we performed, lead SNP identification, annotation and functional mapping with FUMA [29]. Since five analyses were performed, a p-value < 0.01 (genome-wide threshold of $< 1 \cdot 10^{-8}$) was considered statistically significant, other FUMA parameters were left to default settings ($r^2 \geq 0.6$ to define independent significant SNVs and $r^2 \geq 0.1$ to define lead SNVs with a maximum 250 kb distance between LD blocks to merge into a locus). Linkage disequilibrium R^2 values of pairs of variants were looked up with the LDpair Tool [30] using the British in England and Scotland population.

Table S7 Sensitivity analysis: Number of subtype positive cases at different thresholds. Participants were considered subtype positive when the posterior average loading exceeded the specified threshold.

Threshold	B_2	B_3	B_{16}	B_{22}	B_{28}	$B_2 + B_3 + B_{16} + B_{22} + B_{28}$
30%	3,345 (2.74%)	3,716 (3.05%)	4,316 (3.54%)	3,124 (2.56%)	3,498 (2.87%)	20,011 (16.41%)
40%	2,951 (2.42%)	2,892 (2.37%)	3,636 (2.98%)	2,386 (1.96%)	2,363 (1.94%)	16,705 (13.70%)
50%	2,663 (2.18%)	2,286 (1.87%)	3,003 (2.46%)	1,843 (1.51%)	1,541 (1.26%)	13,987 (11.47%)

Table S8 Genome-wide significant single nucleotide variant leads of model 2 with covariates age, sex (male versus female), and smoking status (current/recent, ex-, and never smokers) and 10 principal components (i.e., *without* factors related to obesity). Lead single nucleotide variants on chromosomes 1, 2, 5–8, and 11 (see Table S9 for the rest). Abbreviations: Chr, chromosome; EA, effect allele; MAF, minor allele frequency; NEA, non-effect allele; Pos: genomic position on the human reference genome GRCh37; rsID, reference single nucleotide variant cluster identifier; SE, standard error.

rsID	Subtype	Chr	Pos	EA/NEA	HGVS	MAF	Slope (SE)	$\log_{10} p$	Nearest gene
rs3768321	B_{28}	1	40035928	G/T	NC_000001.10:g.40035928G>T	0.188	-0.042 (0.007)	-8.22	PABPC4:RP11-69E11.8
rs583104	B_2	1	109821307	G/T	NC_000001.10:g.109821307G>T	0.223	0.058 (0.007)	-15.83	PSRC1
rs1041968	B_{16}	2	21232804	G/A	NC_000002.11:g.21232804G>A	0.442	-0.041 (0.006)	-12.31	APOB
rs533617	B_{16}	2	21233972	C/T	NC_000002.11:g.21233972T>C	0.049	-0.082 (0.012)	-10.17	APOB
rs1260326	B_{16}	2	27730940	C/T	NC_000002.11:g.27730940T>C	0.41	-0.042 (0.006)	-11.67	GCKR
rs13431652	B_{28}	2	169753415	C/T	NC_000002.11:g.169753415T>C	0.311	-0.061 (0.007)	-19.1	SPC25
rs569805	B_2	2	169782880	T/A	NC_000002.11:g.169782880A>T	0.364	0.038 (0.007)	-8.22	ABCB11
rs494874	B_{22}	2	169789306	C/T	NC_000002.11:g.169789306T>C	0.36	0.04 (0.006)	-9.62	ABCB11
rs853789	B_3	2	169801488	G/A	NC_000002.11:g.169801488A>G	0.353	0.041 (0.006)	-11.87	ABCB11
rs2943634	B_{22}	2	227068080	C/A	NC_000002.11:g.227068080A>C	0.348	0.037 (0.006)	-9	AC068138.1
rs71624138	B_{16}	5	55870395	G/A	NC_000005.9:g.55870395G>A	0.128	-0.057 (0.01)	-8.4	AC022431.2
rs6882842	B_2	5	74651909	G/A	NC_000005.9:g.74651909G>A	0.408	0.04 (0.006)	-10.26	HMGCR
rs7451008	B_2	6	20673880	C/T	NC_000006.11:g.20673880T>C	0.28	0.044 (0.007)	-10.33	CDKAL1
rs35261542	B_{16}	6	20675792	C/A	NC_000006.11:g.20675792C>A	0.279	-0.041 (0.006)	-9.81	CDKAL1
	B_{22}	6					-0.042 (0.006)	-10.07	
rs998584	B_{16}	6	43757896	C/A	NC_000006.11:g.43757896C>A	0.496	-0.035 (0.006)	-8.66	VEGFA
rs2971670	B_{22}	7	44226101	C/T	NC_000007.13:g.44226101C>T	0.177	-0.055 (0.008)	-12.37	GCK
	B_{28}						-0.072 (0.008)	-19.82	
rs917793	B_2	7	44245853	T/A	NC_000007.13:g.44245853A>T	0.178	0.06 (0.008)	-12.54	YKT6
	B_3						0.053 (0.008)	-11.15	
rs1306476	B_{22}	7	72868522	G/A	NC_000007.13:g.72868522A>G	0.189	-0.046 (0.008)	-8.82	BAZ1B
rs11974409	B_{16}	7	72989390	G/A	NC_000007.13:g.72989390A>G	0.186	-0.049 (0.007)	-11.17	TBL2
rs11786900	B_2	8	9239958	G/C	NC_000008.10:g.9239958G>C	0.076	-0.073 (0.01)	-12.01	RP11-115J16.1, RP11-115J16.2
rs268	B_2	8	19813529	G/A	NC_000008.10:g.19813529A>G	0.014	0.109 (0.018)	-9.23	LPL
	B_{16}	8					0.101 (0.017)	-8.63	
	B_{22}	8					0.137 (0.017)	-15.11	
rs3735964	B_2	8	19824045	C/A	NC_000008.10:g.19824045C>A	0.135	0.06 (0.01)	-9.46	LPL
rs9644636	B_{22}	8	19824896	G/T	NC_000008.10:g.19824896T>G	0.272	0.039 (0.007)	-8.03	LPL
rs115849089	B_{16}	8	19912370	G/A	NC_000008.10:g.19912370G>A	0.145	0.068 (0.009)	-14.48	AC100802.3
	B_{22}						0.08 (0.009)	-19.64	
rs28601761	B_{16}	8	126500031	G/C	NC_000008.10:g.126500031C>G	0.408	-0.046 (0.006)	-14.87	RP11-136O12.2
	B_{22}						-0.033 (0.006)	-8.02	
rs10830963	B_2	11	92708710	G/C	NC_000011.9:g.92708710C>G	0.288	0.054 (0.007)	-14.6	MTNR1B
	B_3						0.042 (0.006)	-10.26	
	B_{16}						0.046 (0.007)	-11.34	
	B_{22}						0.052 (0.007)	-14.22	
	B_{28}						0.063 (0.007)	-19.96	
rs141414463	B_{16}	11	116598315	C/T	NC_000011.9:g.116598315C>T	0.012	-0.104 (0.018)	-8.41	BUD13
rs964184	B_{16}	11	116648917	G/C	NC_000011.9:g.116648917G>C	0.162	0.116 (0.008)	-41.89	ZNF259
	B_{22}						0.11 (0.008)	-37.38	

S1.5.1 Post GWAS analysis

To explore the functional relevance of our GWAS findings, we integrated our results with publicly available epigenetic data. We downloaded H3K27ac ChIP-seq data, an active enhancer mark, of the ENCODE consortium from the Gene Expression Omnibus (GEO) for six human tissues: liver (sample GSM1112809), ovary (GSM956009), kidney (GSM1112799), adipose (GSM916066), pancreas (GSM906397), and heart

Table S9 Continuation of Table S8: genome-wide association study hits of model 2, with covariates age, sex (male versus female), and smoking status (current/recent, ex-, and never smokers) and 10 principal components (i.e., *without* factors related to obesity). Abbreviations: Chr, chromosome; EA, effected allele; MAF, minor allele frequency; NEA, non-effected allele; Pos: genomic position on human reference genome GRCh37; rsID, reference single nucleotide variant cluster identifier; SE, standard error.

rsID	Subtype	Chr	Pos	EA/NEA	HGVS	MAF	Slope (SE)	$\log_{10} p$	Nearest gene
rs12448528	B_{16}	16	56985555	G/A	NC_000016.9:g.56985555A>G	0.213	-0.042 (0.007)	-8.12	AC012181.1
rs56156922	B_{28}	16	56987369	C/T	NC_000016.9:g.56987369T>C	0.293	-0.042 (0.006)	-10.81	AC012181.1
rs247616	B_{16}	16	56989590	C/T	NC_000016.9:g.56989590C>T	0.292	0.045 (0.006)	-12.83	AC012181.1
rs118146573	B_{22}	16	57000938	G/A	NC_000016.9:g.57000938G>A	0.122	-0.064 (0.009)	-11.88	CETP
rs7205804	B_{22}	16	57004889	G/A	NC_000016.9:g.57004889G>A	0.427	0.059 (0.006)	-23.46	CETP
rs11076175	B_2	16	57006378	G/A	NC_000016.9:g.57006378A>G	0.206	0.049 (0.008)	-8.89	CETP
	B_{16}						0.049 (0.008)	-9.4	
rs2952151	B_2	17	37828496	C/T	NC_000017.10:g.37828496T>C	0.314	-0.041 (0.007)	-8.53	PGAP3
rs8106503	B_2	19	11196886	C/T	NC_000019.9:g.11196886T>C	0.111	0.077 (0.009)	-15.93	LDLR
rs151330717	B_2	19	45196964	G/A	NC_000019.9:g.45196964G>A	0.012	-0.186 (0.025)	-12.74	CTB-171A8.1
rs62120566	B_2	19	45197732	G/C	NC_000019.9:g.45197732C>G	0.015	0.136 (0.023)	-8.77	CTB-171A8.1
rs11881756	B_2	19	45220896	C/T	NC_000019.9:g.45220896T>C	0.102	0.076 (0.011)	-10.59	CTB-171A8.1
rs2965169	B_2	19	45251156	C/A	NC_000019.9:g.45251156A>C	0.437	0.055 (0.007)	-14.85	BCL3
rs2967668	B_2	19	45302951	G/A	NC_000019.9:g.45302951A>G	0.126	0.057 (0.01)	-8.14	CBLC
rs111371860	B_2	19	45345787	T/A	NC_000019.9:g.45345787A>T	0.076	0.139 (0.013)	-24.82	PVRL2
rs4452060	B_2	19	45347911	C/A	NC_000019.9:g.45347911C>A	0.457	0.06 (0.006)	-21.78	PVRL2
rs404935	B_2	19	45372794	G/A	NC_000019.9:g.45372794G>A	0.141	-0.076 (0.008)	-19.55	PVRL2
rs6859	B_2	19	45382034	G/A	NC_000019.9:g.45382034A>G	0.44	0.037 (0.006)	-8.96	PVRL2
rs6857	B_3	19	45392254	C/T	NC_000019.9:g.45392254C>T	0.165	-0.053 (0.007)	-12.74	PVRL2:CTB-129P6.4
	B_{22}						-0.051 (0.008)	-10.92	
rs11668327	B_2	19	45398633	G/C	NC_000019.9:g.45398633G>C	0.156	-0.077 (0.008)	-18.78	TOMM40
rs7412	B_2	19	45412079	C/T	NC_000019.9:g.45412079C>T	0.063	-0.281 (0.011)	-139.31	APOE
rs56131196	B_2	19	45422846	G/A	NC_000019.9:g.45422846G>A	0.198	0.061 (0.008)	-14.83	APOC1
rs4420638	B_{16}	19	45422946	G/A	NC_000019.9:g.45422946A>G	0.198	0.07 (0.007)	-21.23	APOC1
rs117261169	B_2	19	45491032	C/T	NC_000019.9:g.45491032C>T	0.011	-0.221 (0.027)	-15.15	CLPTM1
rs140912273	B_2	19	45507696	G/A	NC_000019.9:g.45507696G>A	0.014	-0.162 (0.026)	-9.5	RELB

Table S10 Per anthropometric feature quantile group, the age summary statistics of participants in that group. Mean: μ; standard deviation: σ; lower quartile: Q_1; median: Q_2; upper quartile: Q_3.

Feature	category	μ	σ	Q_1	Q_2	Q_3
waist-hip-ratio	0-10%	38.87	11.91	29.00	39.00	47.00
	10-30%	43.52	12.27	35.00	44.00	51.00
	30-70%	47.17	12.44	39.00	47.00	55.00
	70-90%	49.95	12.25	42.00	49.00	59.00
	90-100%	51.78	12.03	44.25	51.00	61.00
weight	0-10%	43.55	14.65	31.00	43.00	53.00
	10-30%	45.72	13.67	36.00	46.00	54.00
	30-70%	47.21	12.66	39.00	47.00	55.00
	70-90%	47.68	11.80	40.00	48.00	55.00
	90-100%	46.60	10.81	40.00	46.00	52.00
BMI	underweight	36.50	13.20	26.00	34.00	46.00
	normal	43.92	12.82	34.00	44.00	52.00
	overweight	48.92	12.37	41.00	48.00	57.00
	obesity-CI	49.55	12.02	42.00	49.00	58.00
	obesity-CII	47.88	11.32	41.00	47.00	54.00
	obesity-CIII	46.66	11.43	39.25	46.00	52.00

Table S11 Per blood feature quantile group, the age summary statistics of participants in that group. Mean: μ; standard deviation: σ; lower quartile: Q_1; median: Q_2; upper quartile: Q_3. Abbreviations: ALB, albumin; ALP, alkaline phosphatase; ALT, alanine aminotransferase; AST, aspartate aminotransferase; CHOL, total cholesterol; CREA, creatinine; CRP, C-reactive protein; FIB-4, fibrosis-4 index; GGT, γ-glutamyltransferase; GLU, glucose.

feature	category	μ	σ	Q_1	Q_2	Q_3
ALT	0-10%	43.28	12.72	34.00	43.00	50.00
	10-30%	44.47	12.04	36.00	44.00	51.00
	30-70%	47.00	11.78	40.00	47.00	53.00
	70-90%	48.28	11.73	41.00	48.00	56.00
	90-100%	47.53	11.42	40.00	48.00	54.00
AST	0-10%	43.52	11.01	36.00	44.00	50.00
	10-30%	44.48	11.50	37.00	45.00	51.00
	30-70%	46.32	11.87	39.00	46.00	52.00
	70-90%	48.69	12.31	41.00	49.00	57.00
	90-100%	49.20	12.28	41.00	49.00	57.00
ALP	0-10%	43.84	10.39	38.00	44.00	50.00
	10-30%	44.62	10.97	38.00	45.00	51.00
	30-70%	46.22	11.88	39.00	46.00	52.00
	70-90%	48.49	12.64	40.00	49.00	57.00
	90-100%	49.59	13.10	41.00	50.00	59.00
γ-GT	0-10%	42.59	11.47	35.00	43.00	50.00
	10-30%	44.37	11.63	36.00	44.00	51.00
	30-70%	46.73	12.02	39.00	47.00	53.00
	70-90%	48.33	11.99	41.00	48.00	56.00
	90-100%	49.36	11.41	42.00	49.00	56.00
CRP	0-10%	44.64	11.65	37.00	45.00	51.00
	10-30%	46.48	11.86	39.00	46.00	53.00
	30-70%	47.71	12.53	40.00	48.00	56.00
	70-90%	47.20	13.18	38.00	47.00	55.00
	90-100%	46.58	13.00	38.00	46.00	53.00
CHOL	0-10%	39.70	13.74	29.00	38.00	47.00
	10-30%	41.92	12.53	33.00	41.00	49.00
	30-70%	46.30	11.93	39.00	46.00	52.00
	70-90%	51.20	11.33	44.00	51.00	59.00
	90-100%	54.36	10.68	48.00	54.00	62.00
HDL	0-10%	44.89	12.44	36.00	45.00	52.00
	10-30%	45.43	12.64	37.00	45.00	52.00
	30-70%	46.10	12.82	38.00	46.00	53.00
	70-90%	47.71	12.74	40.00	48.00	56.00
	90-100%	50.17	12.48	43.00	50.00	59.00
LDL	0-10%	40.86	14.15	30.00	39.00	49.00
	10-30%	42.53	12.72	33.00	42.00	50.00
	30-70%	46.26	12.08	39.00	46.00	53.00
	70-90%	50.75	11.50	44.00	50.00	59.00
	90-100%	53.62	10.83	47.00	53.00	62.00
CREA	0-10%	46.46	12.78	38.00	47.00	54.00
	10-30%	45.72	12.57	37.00	46.00	53.00
	30-70%	45.98	12.56	38.00	46.00	53.00
	70-90%	47.09	12.64	39.00	47.00	55.00
	90-100%	50.21	13.68	41.00	49.00	60.00
GLU	0-10%	41.22	12.23	32.00	41.00	49.00
	10-30%	42.89	12.45	34.00	43.00	50.00
	30-70%	46.12	12.29	38.00	46.00	53.00
	70-90%	50.48	11.76	43.00	50.00	59.00
	90-100%	55.17	11.53	47.00	54.00	64.00

(GSM1013124). After download, the `.wig.gz` files were converted to `.bigwig` format using UCSC `wigToBigWig`. Lead single nucleotide variants (SNVs) of GWAS model 1 (Tables 2 & 3, Main Manuscript) and model 2 (Tables S8 & S9) were analyzed separately, and grouped by the five candidate CKM subtypes (B_2, B_3, B_{16}, B_{22}, and B_{28}). As a quality control check, we verified that all datasets had at least 10 million uniquely mapped reads (Table S14), following CHIP-seq guidelines and practices of the ENCODE and modENCODE consortia [31]. Four out of five datasets also pass a more stringent sequencing depth threshold of 16 million reads (in drosophila) [32]. As a secondary quality control measure, we verified that housekeeping gene ACTB is expressed highly in all tissue types, including heart tissue which has the lowest mapped reads amongst the 6 tissues we selected.

After converting lead variants to `.bed` files, we used the `deepTools` package [33] to process and visualize activity 5 kb up and downstream of the variants, with a bin size of 10 basepairs, and imputing missing values with zeroes (Figs. S8 & S9).

To identify biological pathways and functions enriched among the identified genetic associations, we performed an over representation analysis for the set of nearest genes to the lead SNVs in each of the five subtypes (B_2, B_3, B_{16}, B_{22}, and B_{28}). We used the `gseapy` package [34] with the Enrichr platform to query the Kyoto Encyclopedia of Genes and Genomes (KEGG) and Gene Ontology (GO) databases. The top 20 enriched pathways and biological processes were then visualized in Fig. S10.

S1.6 Validation in UK biobank

S1.6.1 Cohort compilation and definitions

Of those who participated in the first or second wave, participants matching any of the following criteria were considered unsuitable for genotype analysis (to avoid systematic bias and population substructure confounding) and were excluded:

- participants with sex chromosome aneuploidy (field id: 22019),
- genotype outliers (field id: 22027)
- where gender (self reported, field id: 31) and sex (biological, field id: 22001) did not match, indicating potential mislabelling,
- not of white British ancestry (field id: 22006), to reduce population stratification,
- 1st degree relatives (which were not included in the principal component construction, field id: 22020) to reduce shared household environment confounding,
- or with ten or more 3rd degree relatives (field id: 22021).

Next, diagnosis codes and dates were extracted from in-hospital records (field id: 41270 and 41280 for ICD-10 codes; and field id: 41271 and 41281 for ICD-9 codes) and the cause of death (both the primary cause of death, field IDs: 40000 and 40001, as well as secondary causes of death, field ID: 40002). These were combined with self-reported medication usage (field ID: 20003) to indicate the presence of cardiovascular disease, kidney disease, and type-II diabetes, at any point during their life using indications and contra-indications detailed in Table S15. Cardiovascular disease was defined as the union of heart failure, weak heart, a heart attack, (chronic) reduced heart flow,

chest pain or irregular heart beat. Kidney disease was defined as the union (non-) severe chronic kidney disease and kidney failure.

S1.6.2 Polygenic risk score association

Imputed genotypes (field ID: 21008) were quality controlled using the `bgens_qc` workflow from UK biobank, by downloading and compiling the workflow file https://github.com/dnanexus/UKB_RAP/raw/refs/heads/main/end_to_end_gwas_phewas/bgens_qc/bgens_qc.wdl using the DNAnexus compiler version 2.13.0. The following `plink2_options` settings were used for the workflow: `--hwe 1e-15 --mind 0.1 --geno 0.1 --freq counts --pgen-info`. Generated list of samples and variants that passed quality control were subsequently used to generate two sets of polygenic risk scores using the C+T approach (clumping and thresholding) [35]: one set for model 1 (based on the already thresholded and clumped summary statistics in Tables 2 & 3, Main Manuscript, which came from a GWAS that was adjusted for factors related to obesity), and one for model 2 (Tables S8 and S9, also already thresholded and clumped; generateing using a GWAS without adjustment of factors related to obesity) using the `plink --score` option. Next, scores were centred and standardized to z-scores and combined with 10 principal components and logistically regressed on each of the three diseases separately; $p < 0.05$ was considered statistically significant.

References

[1] Scholtens S, Smidt N, Swertz MA, Bakker SJ, Dotinga A, Vonk JM, et al. Cohort Profile: LifeLines, a three-generation cohort study and biobank. International journal of epidemiology. 2015;44(4):1172–1180.

[2] Niebuur J, Vonk JM, Du Y, de Bock GH, Lunter G, Krabbe PF, et al. Lifestyle factors related to prevalent chronic disease multimorbidity: A population-based cross-sectional study. PLoS One. 2023;18(7):e0287263.

[3] van der Ende MY, Hartman MH, Hagemeijer Y, Meems LM, de Vries HS, Stolk RP, et al. The LifeLines Cohort Study: prevalence and treatment of cardiovascular disease and risk factors. International journal of cardiology. 2017;228:495–500.

[4] Sterling RK, Lissen E, Clumeck N, Sola R, Correa MC, Montaner J, et al. Development of a simple noninvasive index to predict significant fibrosis in patients with HIV/HCV coinfection. Hepatology. 2006;43(6):1317–1325.

[5] Manabe I. Chronic inflammation links cardiovascular, metabolic and renal diseases. Circulation Journal. 2011;75(12):2739–2748.

[6] Chaudhary K, Malhotra K, Sowers J, Aroor A. Uric acid-key ingredient in the recipe for cardiorenal metabolic syndrome. Cardiorenal medicine. 2013;3(3):208–220.

[7] Bhale AS, Venkataraman K. Leveraging knowledge of HDLs major protein ApoA1: Structure, function, mutations, and potential therapeutics. Biomedicine & Pharmacotherapy. 2022;154:113634.

[8] Ferrannini G, Rosenthal N, Hansen MK, Ferrannini E. Liver function markers predict cardiovascular and renal outcomes in the CANVAS Program. Cardiovascular Diabetology. 2022;21(1):127.

[9] ElSayed NA, Aleppo G, Aroda VR, Bannuru RR, Brown FM, Bruemmer D, et al. 11. Chronic Kidney Disease and Risk Management: Standards of Care in Diabetes—2023. Diabetes Care. 2022 12;46(Supplement_1):S191–S202. https://doi.org/10.2337/dc23-S011.

[10] Marassi M, Fadini GP. The cardio-renal-metabolic connection: a review of the evidence. Cardiovascular Diabetology. 2023;22(1):195.

[11] Ndumele CE, Rangaswami J, Chow SL, Neeland IJ, Tuttle KR, Khan SS, et al. Cardiovascular-kidney-metabolic health: a presidential advisory from the American Heart Association. Circulation. 2023;148(20):1606–1635.

[12] Consultation WE. Waist circumference and waist-hip ratio. Report of a WHO Expert Consultation Geneva: World Health Organization. 2008;2008:8–11.

[13] Cappuccio FP, Miller MA. Sleep and cardio-metabolic disease. Current cardiology reports. 2017;19:1–9.

[14] McEvoy JW, McCarthy CP, Bruno RM, Brouwers S, Canavan MD, Ceconi C, et al. 2024 ESC Guidelines for the management of elevated blood pressure and hypertension: Developed by the task force on the management of elevated blood pressure and hypertension of the European Society of Cardiology (ESC) and endorsed by the European Society of Endocrinology (ESE) and the European Stroke Organisation (ESO). European Heart Journal. 2024;p. ehae178.

[15] Erosheva E, Fienberg S, Lafferty J. Mixed-membership models of scientific publications. Proceedings of the National Academy of Sciences. 2004;101(suppl_1):5220–5227.

[16] Lu HM, Wei CP, Hsiao FY. Modeling healthcare data using multiple-channel latent Dirichlet allocation. Journal of biomedical informatics. 2016;60:210–223.

[17] Neijzen D, Donker HC, Vonk J, Lunter G. Unsupervised Learning in Heterogeneous Tabular Data: Application to a Respiratory Disease Cohort. Available at SSRN 5372663. 2025;https://doi.org/10.2139/ssrn.5372663.

[18] Gelman A, Carlin JB, Stern HS, Dunson DB, Vehtari A, Rubin DB. Bayesian Data Analysis. New York: Chapman and Hall/CRC; 2013.

[19] Murphy KP. Probabilistic machine learning: Advanced topics. MIT press; 2023.

[20] Crouse DF. On implementing 2D rectangular assignment algorithms. IEEE T Aero Elec Sys. 2016;52(4):1679–1696.

[21] Rousseeuw PJ. Silhouettes: a graphical aid to the interpretation and validation of cluster analysis. J Comput Appl Math. 1987;20:53–65.

[22] of the Netherlands Consortium TG. Whole-genome sequence variation, population structure and demographic history of the Dutch population. Nature genetics. 2014;46(8):818–825.

[23] Howie BN, Donnelly P, Marchini J. A flexible and accurate genotype imputation method for the next generation of genome-wide association studies. PLoS genetics. 2009;5(6):e1000529.

[24] : http://www.haplotypereference-consortium.org.

[25] Chang CC, Chow CC, Tellier LC, Vattikuti S, Purcell SM, Lee JJ. Second-generation PLINK: rising to the challenge of larger and richer datasets. Gigascience. 2015;4(1):s13742–015.

[26] Mbatchou J, Barnard L, Backman J, Marcketta A, Kosmicki JA, Ziyatdinov A, et al. Computationally efficient whole-genome regression for quantitative and binary traits. Nature genetics. 2021;53(7):1097–1103.

[27] Bulik-Sullivan BK, Loh PR, Finucane HK, Ripke S, Yang J, of the Psychiatric Genomics Consortium SWG, et al. LD Score regression distinguishes confounding from polygenicity in genome-wide association studies. Nature genetics. 2015;47(3):291–295.

[28] Willer CJ, Li Y, Abecasis GR. METAL: fast and efficient meta-analysis of genomewide association scans. Bioinformatics. 2010;26(17):2190–2191.

[29] Watanabe K, Taskesen E, Van Bochoven A, Posthuma D. Functional mapping and annotation of genetic associations with FUMA. Nature communications. 2017;8(1):1826.

[30] Machiela MJ, Chanock SJ. LDlink: a web-based application for exploring population-specific haplotype structure and linking correlated alleles of possible functional variants. Bioinformatics. 2015;31(21):3555–3557.

[31] Landt SG, Marinov GK, Kundaje A, Kheradpour P, Pauli F, Batzoglou S, et al. ChIP-seq guidelines and practices of the ENCODE and modENCODE consortia. Genome research. 2012;22(9):1813–1831.

[32] Jung YL, Luquette LJ, Ho JW, Ferrari F, Tolstorukov M, Minoda A, et al. Impact of sequencing depth in ChIP-seq experiments. Nucleic acids research.

2014;42(9):e74–e74.

[33] Ramírez F, Ryan DP, Grüning B, Bhardwaj V, Kilpert F, Richter AS, et al. deepTools2: a next generation web server for deep-sequencing data analysis. Nucleic acids research. 2016;44(Web Server issue):W160.

[34] Fang Z, Liu X, Peltz G. GSEApy: a comprehensive package for performing gene set enrichment analysis in Python. Bioinformatics. 2023;39(1):btac757.

[35] Choi SW, Mak TSH, O'Reilly PF. Tutorial: a guide to performing polygenic risk score analyses. Nature protocols. 2020;15(9):2759–2772.

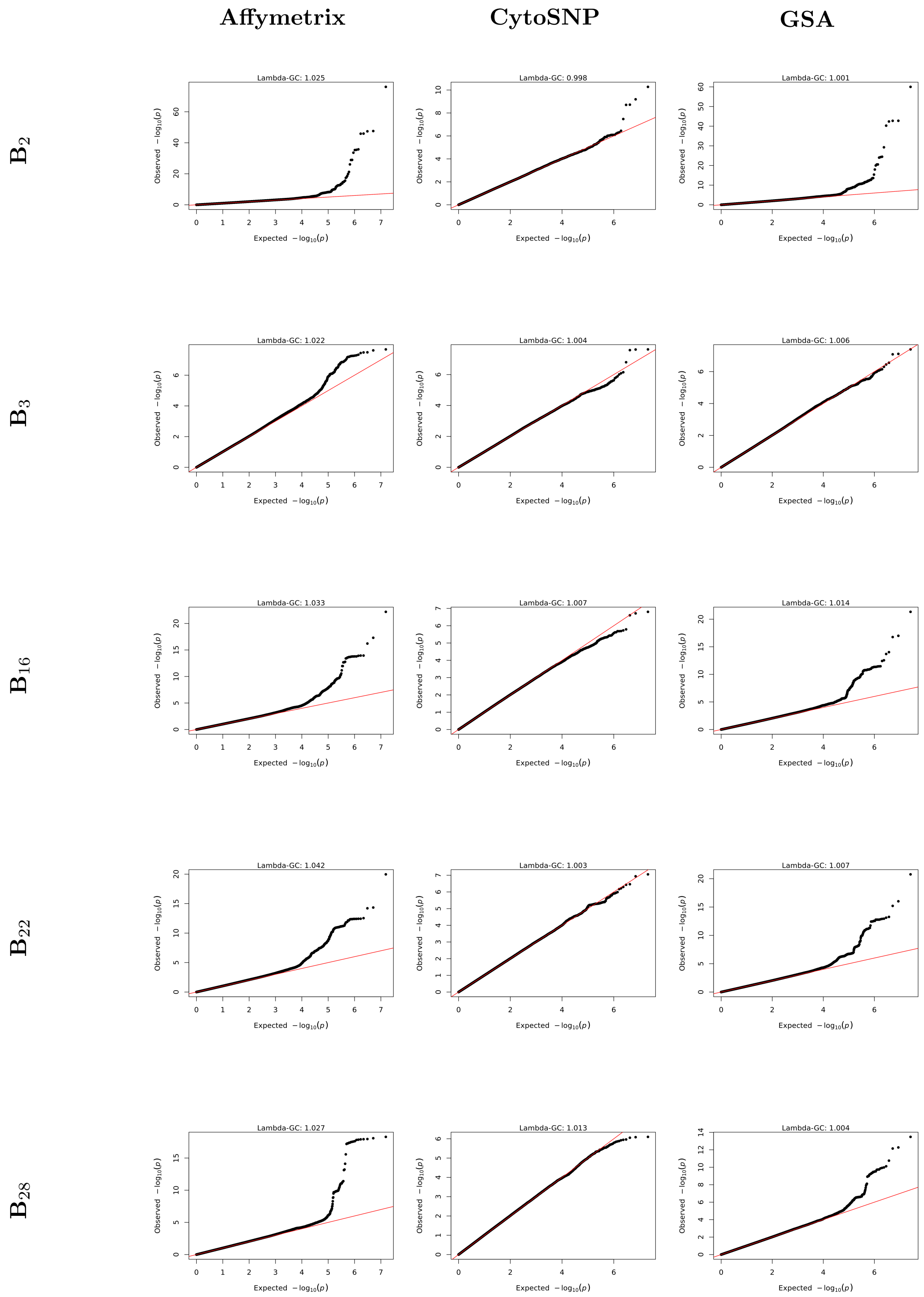

Fig. S4 Quantile-quantile plots and genomic inflation factor λ_{GC} for different candidate subtypes across genotyping platforms. Rows represent candidate CKM subtypes: B_2, B_3, B_{16}, B_{22}, and B_{28}. Columns refer to genotyping platforms: Affymetrix, CytoSNP, and GSA. All analyses were adjusted for age, sex (male versus female), smoking status (current/recent, ex-, and never smokers), body mass index, waist-to-hip ratio, and weight.

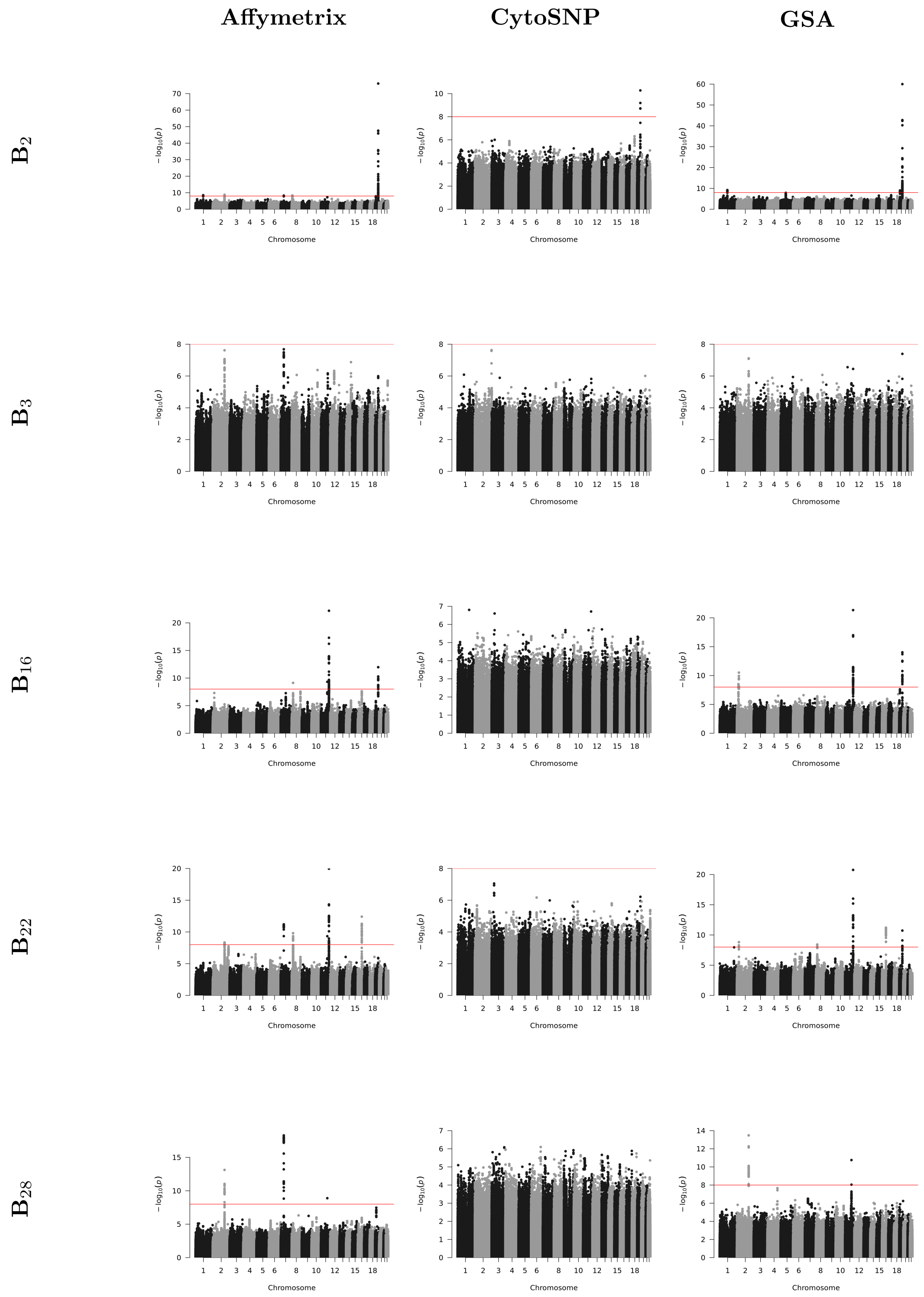

Fig. S5 Manhattan plots for different phenotypes across genotyping platforms. Rows represent candidate subtypes: B_2, B_3, B_{16}, B_{22}, and B_{28}. Columns refer to genotyping platforms: Affymetrix, CytoSNP, and GSA. All analyses were adjusted for age, sex (male versus female), smoking status (current/recent, ex-, and never smokers), body mass index, waist-to-hip ratio, and weight.

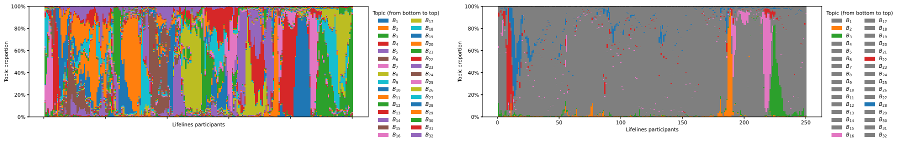

Fig. S6 (a) Posterior average of topic proportions $\boldsymbol{h}^{(i)}$ (y-axis) of a subset of $i = 1, \ldots, 250$ participants (x-axis). Participants have been sorted by solving the travelling sales man problem on the Jensen-Shannon distance between topic proportions. (b) Same as panel (a), but with the candidate CKM profiles highlighted.

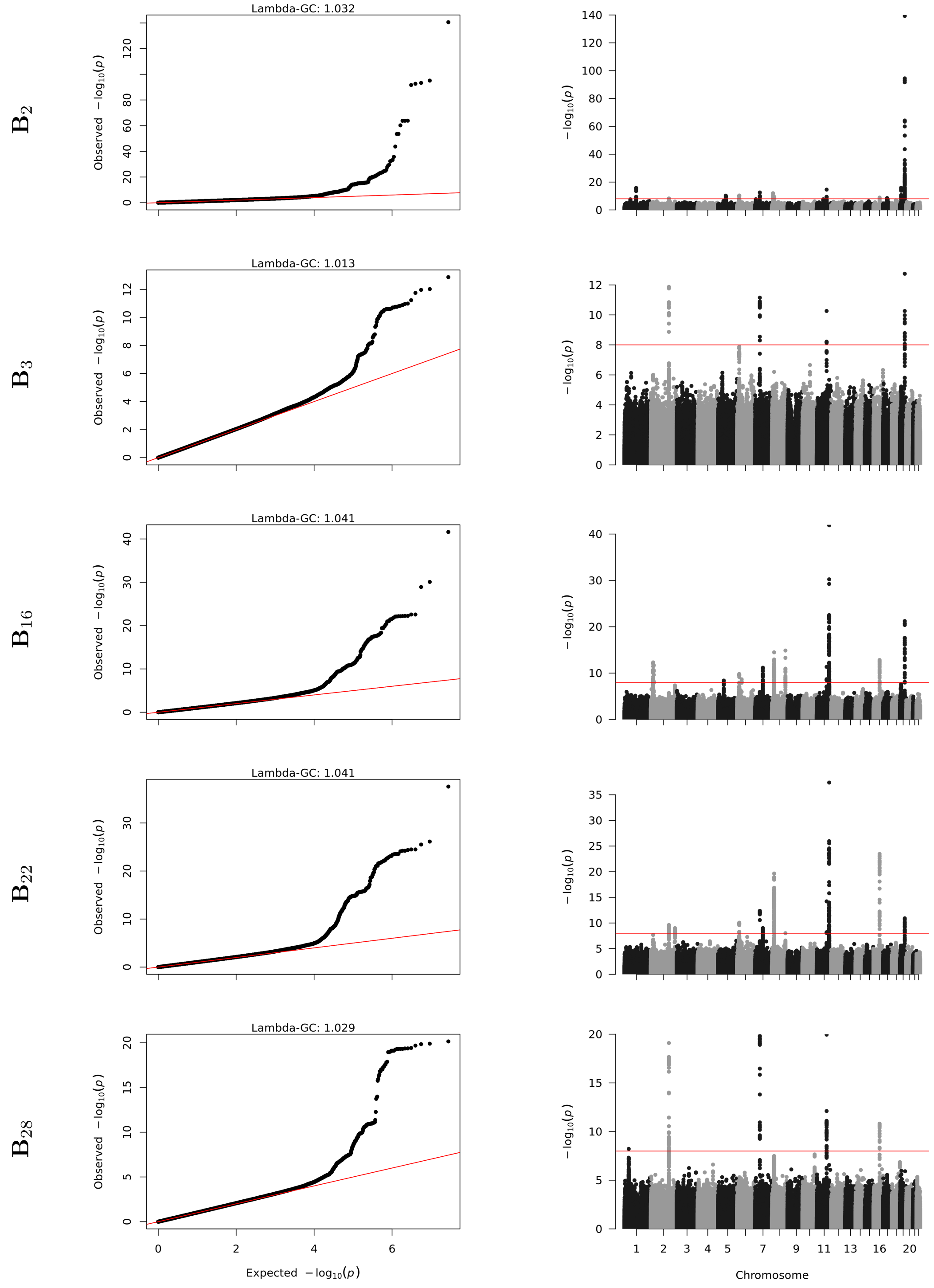

Fig. S7 Meta-analysis of common genetic variants of unrelated individuals from three genotype chips and their relation with five candidate CKM subtypes (B_2, B_3, B_{16}, B_{22}, and B_{28}) *without* adjusting for body mass index, waist-to-hip ratio, and weight [only age, sex (male versus female), and smoking status (current/recent, ex-, and never smokers) was adjusted for]. The quantile-quantile plots (and genomic inflation factor λ_{GC}) show no clear signs of p-value inflation (left) and Manhattan plots show significant hits in strong linkage-disequilibrium (right) indicative of biology, rather than technical artifacts.

Table S12 Continuation of Table S11. Abbreviations: APOA1, apolipoprotein a1; HBA1C, glycated haemoglobin; HDL, high density lipoprotein cholesterol; LDL, low density lipoprotein cholesterol; TRIG, triglyceride; UA, uric acid.

feature	category	μ	σ	Q_1	Q_2	Q_3
HBA1C	0-10%	37.95	11.29	29.00	38.00	46.00
	10-30%	41.09	11.73	32.00	41.00	49.00
	30-70%	45.71	12.06	38.00	46.00	52.00
	70-90%	52.01	11.76	44.00	51.00	61.00
	90-100%	57.29	11.35	49.00	58.00	66.00
APOA1	0-10%	43.02	11.85	34.00	42.00	50.00
	10-30%	45.08	12.44	37.00	45.00	52.00
	30-70%	46.95	12.47	39.00	47.00	54.00
	70-90%	48.97	12.27	42.00	49.00	57.00
	90-100%	49.73	12.42	42.00	50.00	58.00
TRIG	0-10%	42.02	11.65	34.00	42.00	49.00
	10-30%	44.67	12.53	36.00	45.00	52.00
	30-70%	46.96	12.93	38.00	47.00	55.00
	70-90%	48.54	12.83	41.00	48.00	57.00
	90-100%	49.91	11.82	43.00	50.00	58.00
UA	0-10%	45.88	10.90	40.00	46.00	51.00
	10-30%	45.48	11.31	38.00	46.00	52.00
	30-70%	45.63	11.82	38.00	46.00	52.00
	70-90%	47.10	12.46	39.00	47.00	54.00
	90-100%	50.77	12.86	42.00	50.00	60.00
UREA	0-10%	39.97	10.60	31.00	41.00	47.00
	10-30%	42.54	10.79	35.00	43.00	49.00
	30-70%	45.92	11.25	39.00	46.00	52.00
	70-90%	50.15	11.55	43.00	50.00	58.00
	90-100%	55.03	12.00	47.00	54.00	64.00
FIB-4	0-10%	31.42	8.19	25.00	30.00	37.00
	10-30%	39.36	8.38	33.00	40.00	45.00
	30-70%	46.41	8.26	41.00	47.00	51.00
	70-90%	53.97	9.38	48.00	52.00	61.00
	90-100%	61.78	10.58	54.00	63.00	69.00
pressure	normal	41.85	12.37	32.00	42.00	50.00
	elevated	46.39	12.07	39.00	47.00	53.00
	hypertension	53.73	12.35	46.00	52.00	63.00

Table S13 Per urine feature quantile group, the age summary statistics of participants in that group. ALB: albumin; CREA: creatinine; Mean: μ; standard deviation: σ; lower quartile: Q_1; median: Q_2; upper quartile: Q_3.

feature	category	μ	σ	Q_1	Q_2	Q_3
ALB	0-10%	49.16	11.43	42.00	49.00	55.00
	10-30%	48.41	11.87	41.00	48.00	55.00
	30-70%	46.01	11.59	39.00	46.00	52.00
	70-90%	44.12	11.85	36.00	44.00	51.00
	90-100%	45.43	13.36	36.00	46.00	52.00
CREA	0-10%	55.94	12.07	48.00	55.00	65.00
	10-30%	50.56	11.67	43.00	50.00	59.00
	30-70%	46.13	10.96	39.00	47.00	52.00
	70-90%	41.88	10.00	35.00	42.00	49.00
	90-100%	37.68	9.43	30.00	38.00	45.00

Table S14 Sequencing depth of H3K27ac epigenetic markers across six organs. Abbreviations: GEO, Gene Expression Omnibus identified.

Organ	GEO	Mapped reads
Liver	GSM1112809	17,017,206
Ovary	GSM956009	16,625,765
Kidney	GSM1112799	16,263,008
Adipose	GSM916066	16,591,902
Pancreas	GSM906397	12,025,083
Heart	GSM1013124	11,040,740

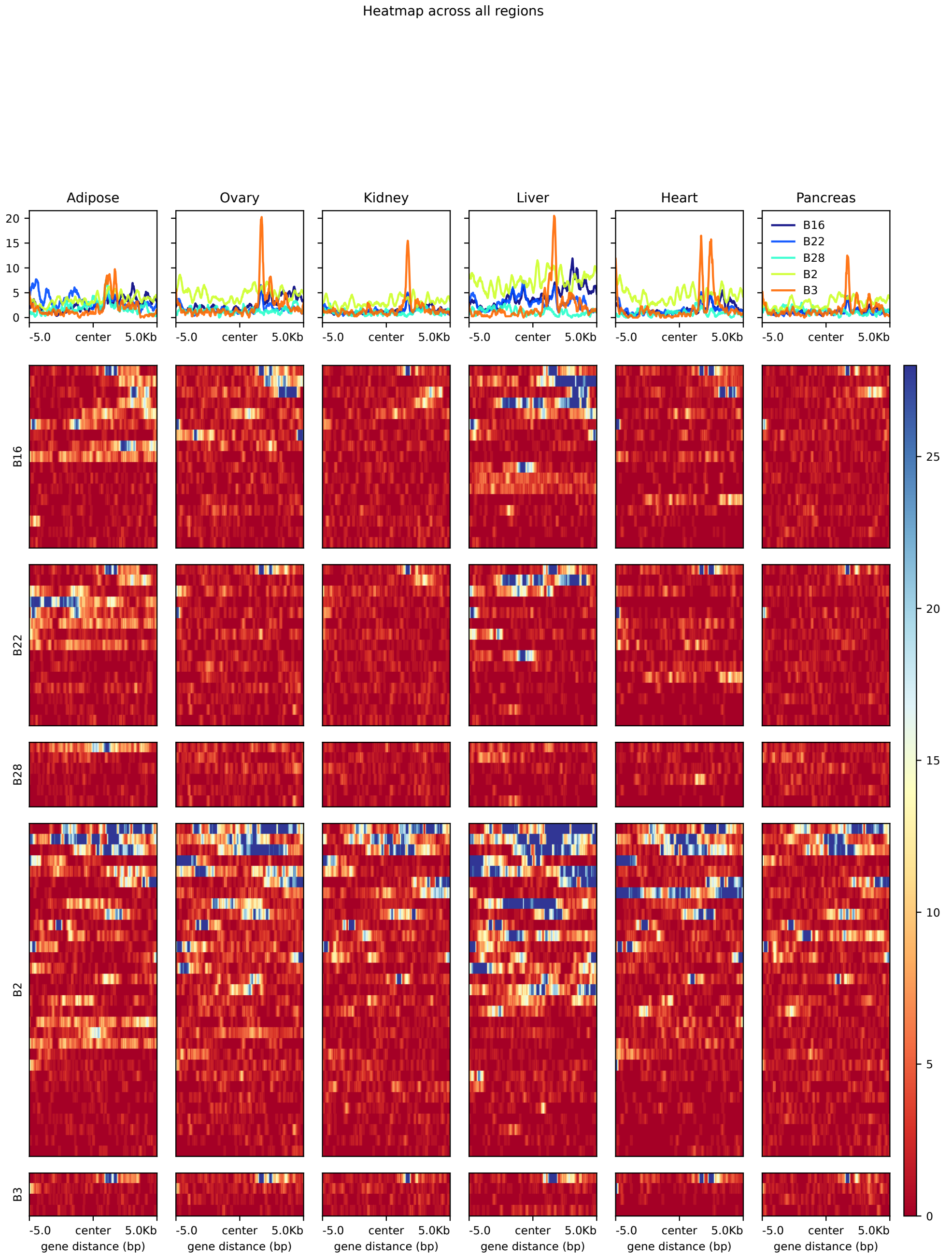

Fig. S8 Per candidate subtype (rows), amount of H3K27Ac histone modification ±5 kb around the lead single nucleotide variants of genome-wide association study model 1 (Tables 2 & 3, Main Manuscript), grouped per tissue type (columns).

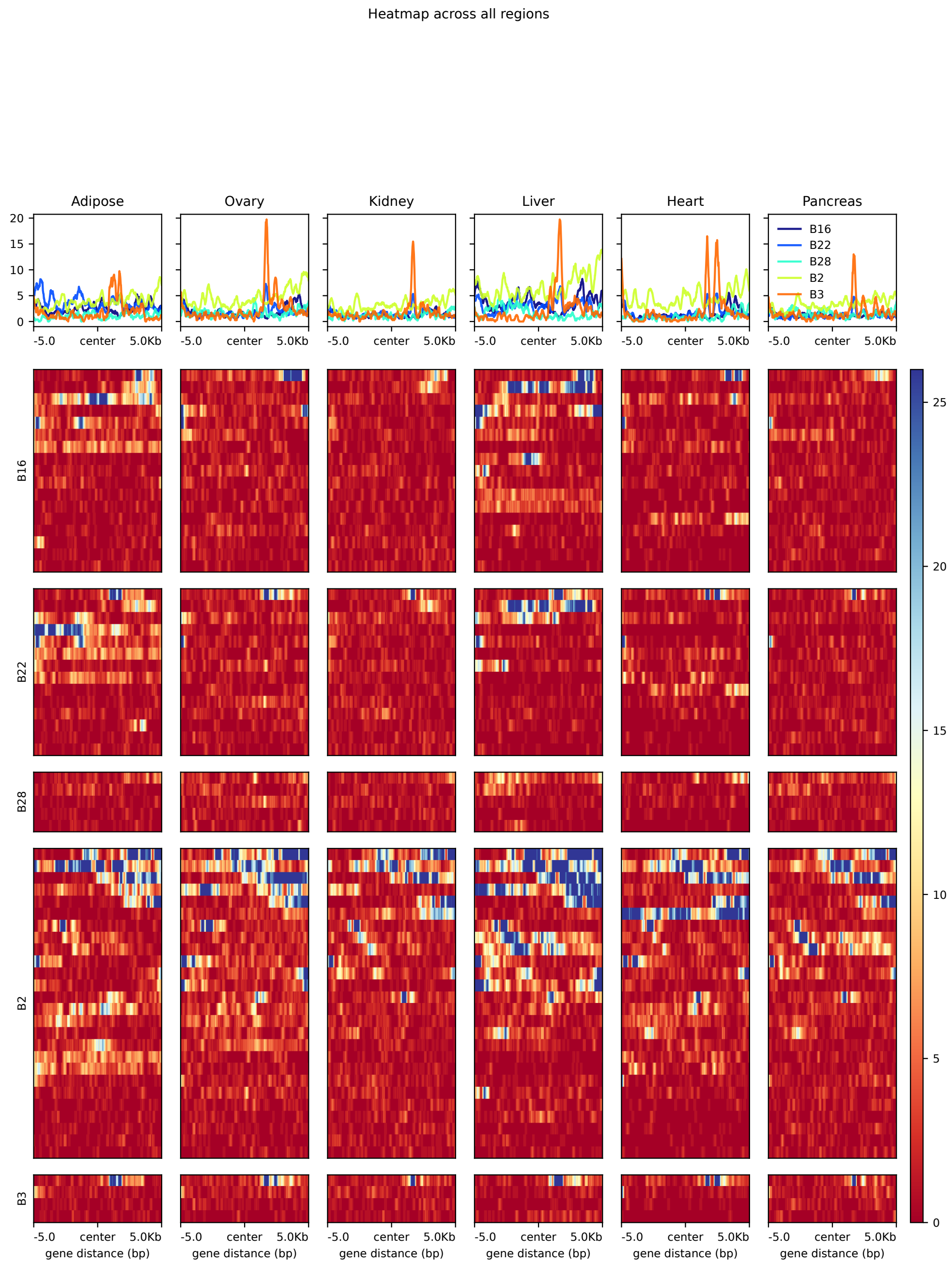

Fig. S9 Like Fig. S8 but for the GWAS hits of model 2 (without adjusting for obesity related factors; Tables S8 & S9).

Model 1
(Adjusted for obesity factors)

Model 2
(Not adjusted for obesity factors)

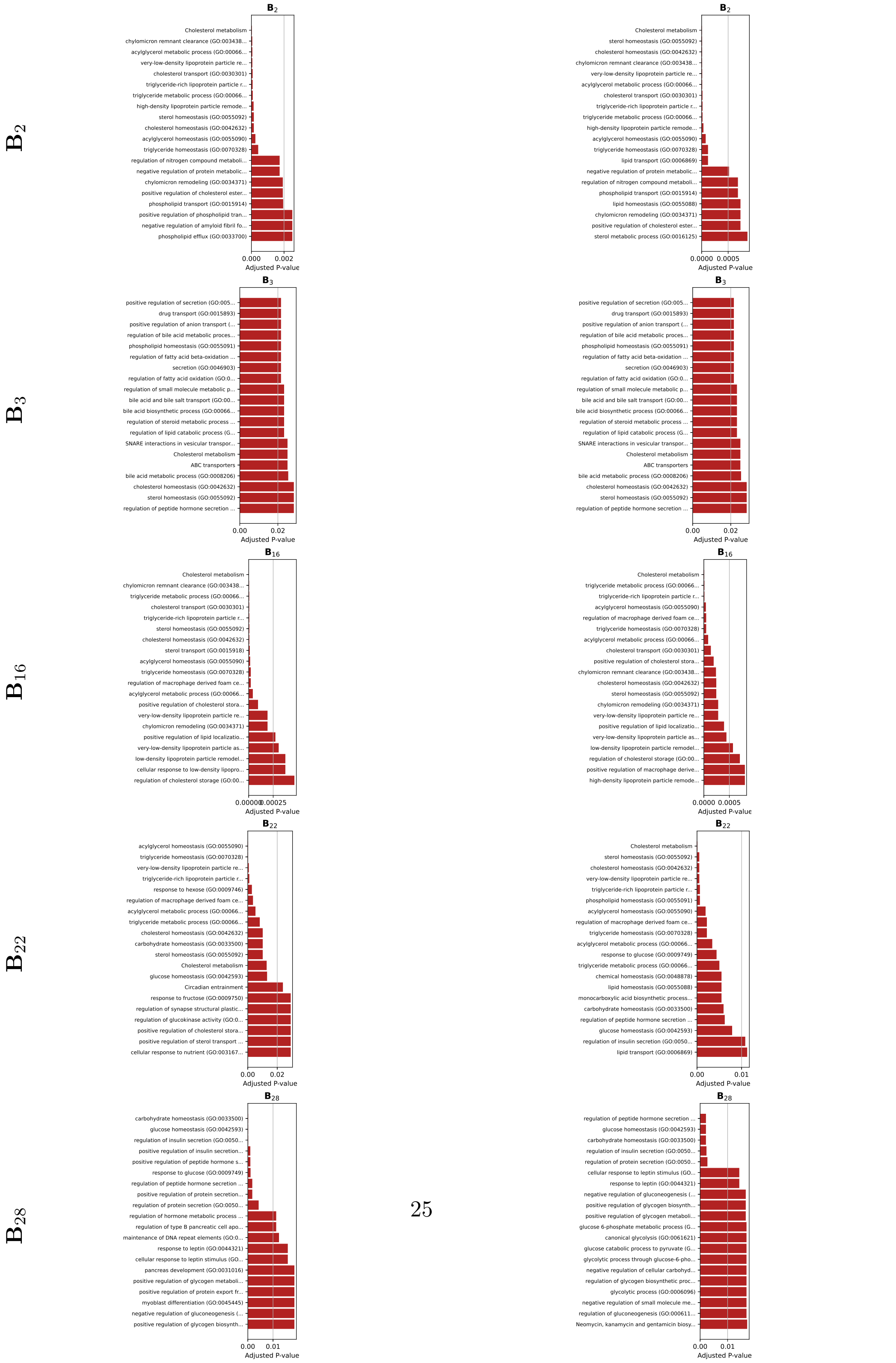

Fig. S10 Top 20 overrepresented pathways in Kyoto Encyclopedia of Genes and Genomes (KEGG) and Gene Ontology (GO) for five candidate subtypes. The analysis is based on the nearest genes to the lead single nucleotide variants from two genome-wide association studies. **Model 1** (left column) was adjusted for demographics, population stratification, and obesity-related factors (Tables 2 & 3, Main Manuscript). **Model 2** (right column) was not adjusted for obesity-related factors (Tables S8 & S9).

Table S15 Disease diagnosis definitions used in data from UK biobank. Abbreviations: ICD-9, international classification of diseases ninth edition; ICD-10, international classification of diseases tenth edition.

**Diagnosis (medical term)**	**Indication / contra indication**	**Hospital discharge*, primary cause of death†, or secondary cause of death‡**		**Medication§**
		**ICD-10**	**ICD-9**	
Heart failure	Indication	I11.0, I13.0, I13.2, I50	4280, 4281, 4289	Furosemide (1140909708) or triamterene + furosemide (1141195254)
Weak heart (cardiomyopathy)	Indication	I42	425	
Heart attack (myocardial infarction)	Indication	I21, I22	410	
	Contra-indication	I21.90, I21.97, I22.90, I22.97		
Heart attack complications (complications following myocardial infarction)	Indication	I23	4296, 4297	
Reduced heart flow (ischemic heart disease)	Indication	I24	4118, 4119	
Chronic heart flow reduction (chronic ischemic heart disease)	Indication	I25	4141, 4148, 4149	
Chest pain (angina pectoris)	Indication	I20	413	
Irregular heart beat (atrial fibrillation and flutter)	Indication	I48	4273	
Chronic kidney disease, non-severe	Indication	N18.1, N18.2, N18.9	585, 5859	
Chronic kidney disease, severe	Indication	N18.3, N18.4	-	
Kidney failure (acute renal failure)	Indication	N17	5845,5849	
Type-2 diabetes	Indication	E11	250	
	Contra-indication	K85.0-K85.9, K86.1, K86.2	5770,5771	

* The following UK biobank field ID's were used. Hospital discharge code/diagnosis date: ICD-10, 41270/41280; ICD-9, 41271/41281. Date of death: 40000.

† Primary cause of death: UK biobank field ID 40001 (ICD-10 only).

‡ Secondary cause of death: UK biobank field ID 40002 (ICD-10 only).

§ Self-reported medication usage: UK biobank field ID 20003. Indicated codes refer to the UK biobank data-coding 4 scheme.

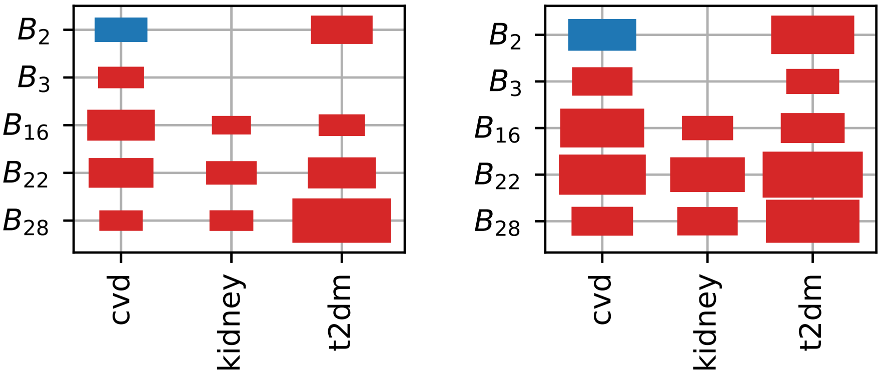

a PRS GWAS model 1 (Tables 2 & 3, Main Manuscript)

b PRS GWAS model 2 (Tables S8 & S9

Fig. S11 Polygenic risk score (PRS) associations with disease diagnoses in UK biobank independently validates three out of five profiles (B_{16}, B_{22}, B_{28}) as *bona fide* CKM subtypes. Logistic regression between PRS of candidate subtypes and 10 principal components (input) and the presence of diseases (output). GWAS model 1 is adjusted for obesity-related factors (see Sec. S1.5 for model details). The size of the red (blue) square scales $\propto \sqrt{|w|}$ with regression weights $\boldsymbol{w}$ and marks a positive (negative) statistical association (t-test at significance level $\alpha = 0.05$, sample size $N = 333,938$). Abbreviations: GWAS, genome-wide association study; PRS, polygenic risk score.
